# Dual inhibitors of DNMT and HDAC induce viral mimicry to induce antitumour immunity in breast cancer

**DOI:** 10.1038/s41420-024-01895-7

**Published:** 2024-03-15

**Authors:** Wenjun Huang, Qingyun Zhu, Zhichao Shi, Yao Tu, Qinyuan Li, Wenwen Zheng, Zigao Yuan, Lulu Li, Xuyu Zu, Yue Hao, Bizhu Chu, Yuyang Jiang

**Affiliations:** 1 School of Pharmacy, Shenzhen University Medical School, Shenzhen University, Shenzhen, 518055 China; 2 The First Affiliated Hospital, Cancer Research Institute, Hengyang Medical School, University of South China, Hengyang, 421001 China; 3 Institute of Biomedical Health Technology and Engineering, Shenzhen Bay Laboratory, Shenzhen, 518132 China; 4 State Key Laboratory of Chemical Oncogenomics, Tsinghua Shenzhen International Graduate School, Shenzhen, 518055 China; 5 School of Pharmaceutical Sciences, Tsinghua University, Beijing, 100084 China

**Keywords:** Breast cancer, Drug development

## Abstract

The existing conventional treatments for breast cancer, including immune checkpoint blockade, exhibit limited effects in some cancers, particularly triple-negative breast cancer. Epigenetic alterations, specifically DNMT and HDAC alterations, are implicated in breast cancer pathogenesis. We demonstrated that DNMTs and HDACs are overexpressed and positively correlated in breast cancer. The combination of DNMT and HDAC inhibitors has shown synergistic antitumour effects, and our previously designed dual DNMT and HDAC inhibitor (termed DNMT/HDACi) 15a potently inhibits breast cancer cell proliferation, migration, and invasion and induces apoptosis in vitro and in vivo. Mechanistically, 15a induces a viral mimicry response by promoting the expression of endogenous retroviral elements in breast cancer cells, thus increasing the intracellular level of double-stranded RNA to activate the RIG-I–MAVS pathway. This in turn promotes the production of interferons and chemokines and augments the expression of interferon-stimulated genes and PD-L1. The combination of 15a and an anti-PD-L1 antibody had an additive effect in vivo. These findings indicate that this DNMT/HDACi has immunomodulatory functions and enhances the effectiveness of immune checkpoint blockade therapy.

**Figure:**
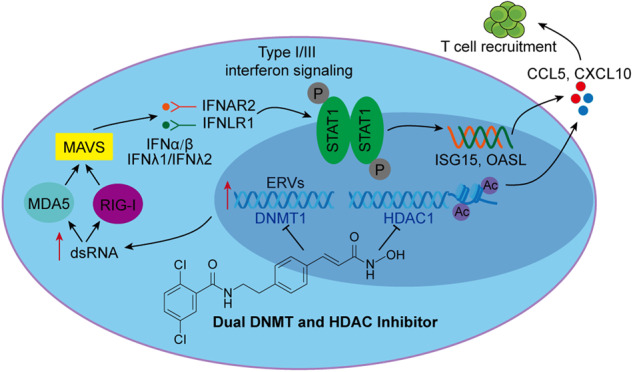
A novel dual DNMT and HDAC inhibitor induces viral mimicry, which induces the accumulation of dsRNA to activate tumoral IFN signalling and cytokine production to enhance the immune response in breast cancer.

A novel dual DNMT and HDAC inhibitor induces viral mimicry, which induces the accumulation of dsRNA to activate tumoral IFN signalling and cytokine production to enhance the immune response in breast cancer.

## Introduction

Although there has been notable progress in the early detection and treatment of breast cancer, the considerable heterogeneity and aggressiveness of the disease make detection and treatment challenging [1–3]. As a result, breast cancer remains the leading cause of cancer-related death in women, second only to lung cancer [4–6]. The adoption of immune checkpoint blockade (ICB) therapy, which has demonstrated early success in treating melanoma and lung cancer, has been relatively slow. Therefore, it is crucial to develop innovative strategies and therapies to improve patient survival rates [7, 8]. Among the recently identified tumour characteristics in 2022, nonmutation epigenetic reprogramming has emerged as a novel feature [9]. Notably, cancer is a complex disease involving both genetic and epigenetic alterations. Epigenetic mechanisms exert crucial influences on multiple aspects of cancer biology, including the promotion of primary tumour growth and invasion, as well as the regulation of immune responses within the tumour microenvironment [10–12]. These mechanisms involve intricate interactions between DNA methylation and histone modification. Among the various classes of enzymes involved in epigenetic regulation, DNA methyltransferase (DNMT) and histone deacetylase (HDAC) have emerged as particularly significant. In fact, targeted drugs, such as the DNMT inhibitor decitabine [13, 14] and the HDAC inhibitor vorinostat (SAHA), have been developed to specifically target these enzymes [15].

Within mammals, there are four distinct members of the DNMT family, namely, DNMT1, DNMT3A, DNMT3B, and DNMT3L [16]. The overexpression of these DNMTs facilitates the methylation of cytosine within CpG islands, thereby leading to the suppression of tumour suppressor genes (TSGs) in human cancer [17]. This phenomenon contributes to the initiation and progression of cancer. Conversely, DNMT inhibitors (DNMTis) reactivate TSGs [18], augment tumour immunogenicity, and induce the secretion of cytotoxic cytokines by various immune cells, including macrophages, natural killer (NK) cells, and CD8^+^ T cells. Ultimately, these actions promote the death of tumour cells. The upregulation of the MHC I class molecules has been observed. Presently, the primary mechanism through which DNMTis exert their immune function is by targeting cancer cells through the induction of viral mimicry. This is achieved by reducing the methylation level of the endogenous retrovirus (ERV) gene promoter and activating the expression of endogenous retroviral elements [19–21]. Consequently, the intracellular levels of double-stranded RNA (dsRNA) are increased, leading to an inflammatory immune response. This response further promotes the production of type I and type III interferons and induces the production of a variety of interferon-stimulated genes (ISGs) and cytokines [19, 22, 23], thereby inhibiting tumour cell immune escape.

HDACs, a group of epigenetic enzymes, are intricately associated with tumorigenesis. In conjunction with histone acetyltransferases (HATs), HDACs govern the equilibrium of histone and protein acetylation through the processes of lysine acetylation and deacetylation [24]. HDACs exert significant regulatory effects on numerous key cellular processes, such as cell proliferation, cellular differentiation, and programmed cell death [25]. Furthermore, the expression and activity of HDACs are frequently disrupted in cancer, whereby HDACs counteract lysine acetylation on histones, thereby inducing chromatin remodelling and altering the expression of TSGs [26, 27], ultimately contributing to increased susceptibility to carcinogenesis. Inhibiting HDACs can result in the release of oncogenic transcription suppressors, leading to cell cycle arrest and apoptosis. Moreover, HDAC inhibitors (HDACis) upregulate the expression of T-cell chemokines, namely, CCL5 and CXCL10, thereby establishing a direct correlation with increased T-cell infiltration within tumours [28, 29]. Conversely, HDACis also decrease the abundance of tumour-associated cells, such as myeloid-derived suppressor cells (MDSCs), and increase the expression of PD-L1 and PD-L2 [30] while concurrently increasing the efficacy of ICB therapies such as PD-1 blockade [31, 32]. These findings substantiate the notion that the combination of HDACis with immunotherapy has the potential to amplify the immunotherapeutic response.

However, the interplay between DNA methylation and histone deacetylation induces a self-reinforcing silencing mechanism that triggers a compensatory resistance mechanism, resulting in suboptimal efficacy of monotherapy and the onset of resistance. Research has demonstrated that HDACis can downregulate the expression of the DNMT1 protein within the nucleus in human breast cancer cells [33], while inhibiting HDAC3 can increase the acetylation level of DNMT1, thereby decreasing its stability. The simultaneous inhibition of HDAC3 and DNMT1 using the BG45 and DNMT1 inhibitors 5-azacytidine (AZA), respectively, has been found to induce synergistic DNMT1 downregulation, growth inhibition and apoptosis in multiple myeloma (MM) cell lines and MM patients [34]. This finding suggested that the combination of DNMTis and HDACis improves breast cancer outcomes and has synergistic effects [35–38]. However, it is important to acknowledge certain significant issues associated with combination therapy, including drug interactions, toxic side effects, and patient adherence.

To address this concern, our laboratory has generated a series of dual inhibitors targeting DNMTs and HDACs. This innovative strategy enables the concurrent modulation of two modifying enzymes, which could alleviate the toxicity associated with the administration of multiple drugs and ultimately increase the efficacy of cancer therapy. Among the scrutinized compounds, 15a is a remarkable candidate. Derived from the DNMT inhibitor NSC-319745, 15a was identified as a potential lead compound that has inhibitory effects on both DNMT and HDAC enzymes [37]. The findings of this study demonstrated that 15a effectively inhibits tumour proliferation both in vitro and in vivo. Additionally, 15a induces a viral mimicry response by inducing the expression of endogenous retroviral elements in breast cancer cells, leading to activation of the RIG-I–MAVS pathway induced by high intracellular levels of dsRNA, which in turn enhances the production of type I/III IFN, the transcription of ISGs and the secretion of chemokines. Moreover, 15a upregulated PD-L1 expression and enhanced anti-PD-L1 antitumour immunity in an immunocompetent mouse model. The primary aim of this study was to elucidate the antitumour mechanism of 15a in breast cancer, providing valuable insights for further pharmacological investigations and enhancing our understanding of the biological activity of dual DNMT/HDAC inhibitors.

## Result

### DNMT1 and HDAC1 are overexpressed and inhibitors of these enzymes exert synergistic effects in human breast cancer

**Fig. 1:**
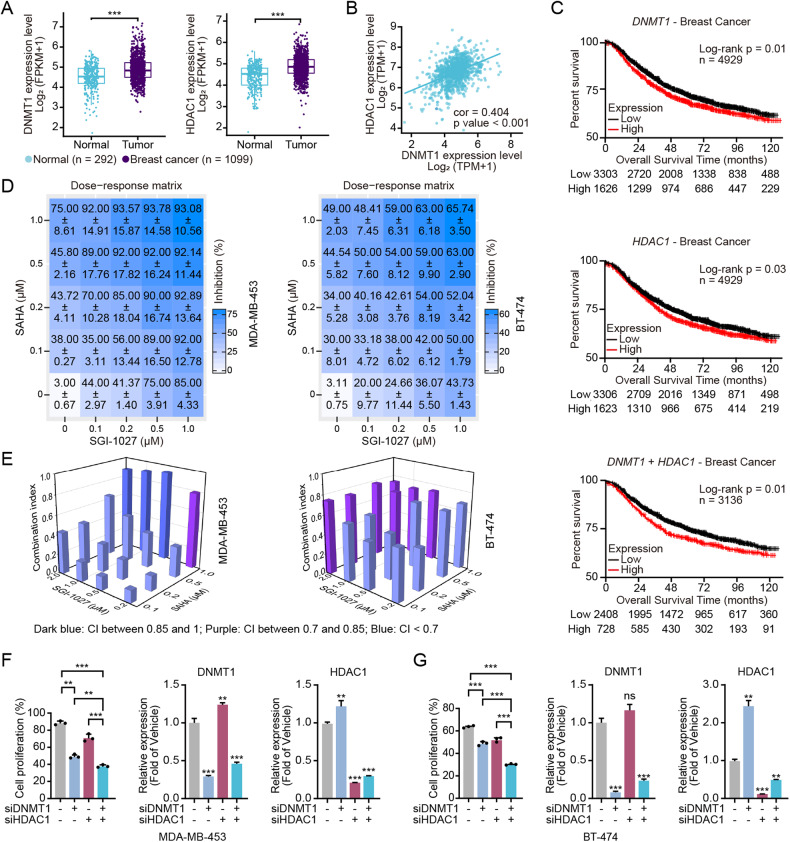
DNMT1 and HDAC1 are overexpressed and inhibitors of these enzymes induce synergistic effects in human breast cancer. **A** Representative box plot generated from the TCGA database showing the relative expression of the DNMT1 and HDAC1 genes in normal and breast cancer tissues. (**B**) Scatter plot showing the correlation between DNMT1 and HDAC1 gene expression levels in breast cancer. **C** Kaplan–Meier plots representing the OS of breast cancer patients in the TCGA dataset categorized according to DNMT1, HDAC1, and the expression of both the DNMT1 and HDAC1 genes. The *p*-value was calculated using a log-rank test. **D** Cell proliferation data for MDA-MB-453 and BT-474 cells treated with DNMTis (SGI-1027) or HDACis (SAHA) alone or in combination at 0.1, 0.2, 0.5 and 1.0 µM each. The data points illustrate the mean of three distinct experiments accompanied by standard deviation (SD) values. **E** Synergistic effects of four different combinations of SGI-1027 and SAHA. A representative example of three different experiments is shown. **F**, **G** MDA-MB-453 and BT-474 cells were transfected with 100 nM siRNA against DNMT1 and HDAC1 for 48 h, and cell viability was assessed using an MTT assay for 72 h. **p < 0.01; ***p < 0.001.

First, we investigated DNMT1 and HDAC1 expression in normal and breast cancer subtypes. The rationale behind selecting these two enzymes as the focus of our research primarily stems from the fact that 15a exhibits greater potency in inhibiting DNMT1 activity than DNMT3A and DNMT3B. Additionally, within the HDAC family, HDAC1 predominantly assumes a pro-cancer function, whereas HDAC6 potentially serves as an anticancer factor [39]. The results showed that DNMT1 and HDAC1 expression was significantly upregulated in breast cancer tissues vs. normal tissues (Fig. 1A). Further detailed analysis revealed that DNMT1 mRNA expression was positively associated with HDAC1 mRNA expression (Fig. 1B). To determine the prognostic value of DNMT1 and HDAC1 in overall survival, Kaplan–Meier survival analysis was performed, and the results showed that high DNMT1 and HDAC1 expression was significantly associated with a poor prognosis (Fig. 1C). Furthermore, to prove that simultaneous inhibition of DNMTs and HDACs could improve cancer treatment efficacy [40], we treated MDA-MB-453 and BT-474 cells with DNMTis (SGI-1027) and HDACis (SAHA). The results showed that the combination of SGI-1027 and SAHA induced greater inhibition of cell proliferation than either inhibitor alone at any tested concentration (Fig. 1D, E). This analysis demonstrated that the combination of these two inhibitors had synergistic effects on reducing the growth of breast cancer cells. We also demonstrated the anticancer effects of knocking down DNMT1, HDAC1 or both, and the results showed that simultaneous inhibition of DNMT1 and HDAC1 induced more potent effects than inhibition of either target alone (Fig. 1F, G). Overall, these findings suggest that DNMTis and HDACis have synergistic antitumour effects in human breast cancer.

### The DNMT/HDACi 15a regulates the activities of DNMT1 and HDAC1 in breast cancer

**Fig. 2:**
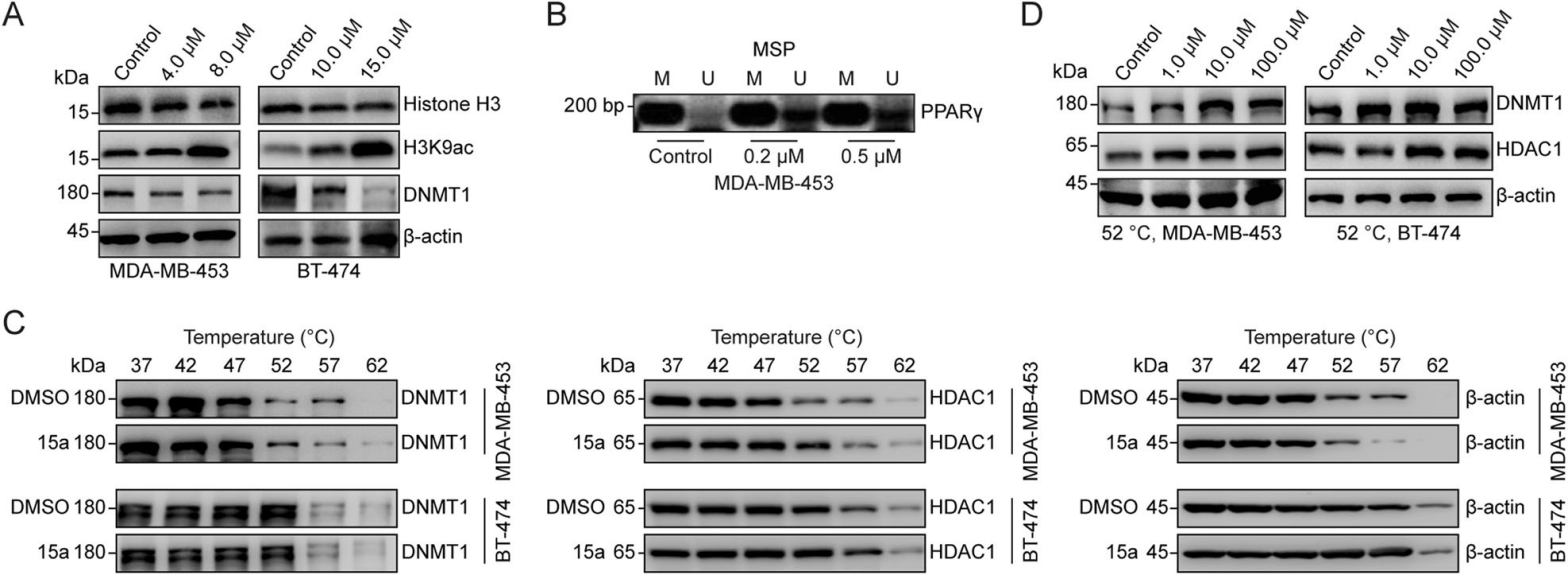
15a regulates the activity of DNMT1 and HDAC1. **A** MDA-MB-453 and BT-474 cells were treated with different concentrations of 15a for 24 h, and the lysates were collected for immunoblotting with the indicated antibodies. **B** Methylation-specific PCR (MSP) analysis of DNA methylation in the PPARr promoter region in MDA-MB-453 cells treated with different concentrations of 15a. The bands in the lanes labelled “M” and “U” are PCR products amplified with primers specific for methylated and unmethylated DNA, respectively. **C** Thermal shift assay (TSA) of DNMT1 and HDAC1 in 15a-treated MDA-MB-453 and BT-474 cells at 10.0 µM; lysates were collected for immunoblotting with the indicated antibodies. **D** TSA of DNMT1 and HDAC1 at different concentrations of 15a were used to treat MDA-MB-453 and BT-474 cells at 52 °C, and the lysates were collected for immunoblotting with the indicated antibodies.

We evaluated the antitumour efficacy of the DNMT/HDACi 15a. SGI-1027 and SAHA as control compounds, and our experiments confirmed that 15a had a weak antiproliferative effect on MCF-7 cells, but for other breast cancer cells, it had good antiproliferative effects (Table 1). Additionally, the proliferation inhibition effects in MDA-MB-453 and 4T1 cells were comparable to in SGI-1027 and SAHA cells (Table 1). To determine the on-target effect of 15a on DNMTs and HDACs, we detected the acetylation level of histone H3 and found that 15a treatment for 24 h induced the upregulation of H3 acetylation in MDA-MB-453 and BT-474 cells (Fig. 2A). We also achieved the same results with other cell lines, such as MDA-MB-231, HCC1937, 4T1 and HCC38 (Fig. S1A). We found that 15a treatment resulted in decreased global DNA demethylation via MSP assay [40] (Fig. 2B) and also found that 15a treatment induced downregulation of DNMT1 protein expression (Fig. 2A). Next, we performed a thermal shift assay (TSA) to confirm that 15a directly binds to the DNMT1 and HDAC1 proteins (Fig. 2C, D), which showed that 15a increased the stability of DNMT1 and HDAC1 under high-temperature conditions in vitro. These data suggested that 15a interacts with DNMT1 and HDAC1 directly.

**Table 1:** Anti-proliferative activity of DNMT/HDAC dual inhibitor 15a in breast cancer cell lines.

Cpds	IC_50_ (µM)		
	15a	Vorinostat	SGI-1027
MDA-MB-436	5.03 ± 0.64	4.01 ± 0.85	1.21 ± 0.04
MDA-MB-231	4.35 ± 0.71	2.08 ± 0.21	0.98 ± 0.12
MDA-MB-468	5.56 ± 0.41	5.01 ± 1.22	0.61 ± 0.20
MDA-MB-361	5.51 ± 1.33	1.59 ± 0.30	1.32 ± 0.62
MDA-MB-453	1.24 ± 0.17	1.16 ± 0.20	0.57 ± 0.12
MDA-MB-157	6.07 ± 0.78	2.07 ± 0.11	0.63 ± 0.10
BT-549	8.03 ± 1.17	7.52 ± 2.37	1.01 ± 0.34
BT-474	8.82 ± 1.24	1.79 ± 0.45	2.09 ± 0.21
HCC1937	9.19 ± 0.45	10.27 ± 3.56	0.94 ± 0.07
HCC38	2.11 ± 0.65	0.86 ± 0.06	0.59 ± 0.08
HCC1143	4.85 ± 1.59	1.90 ± 1.18	1.42 ± 0.18
T-47D	8.29 ± 3.22	2.82 ± 1.41	0.96 ± 0.53
MCF-7	>25.00	19.30 ± 4.71	1.82 ± 0.42
4T1	2.06 ± 0.84	1.67 ± 0.75	1.24 ± 0.77

Data are expressed as the mean ± SD from the dose-response curves of at least three independent experiments.

### The DNMT/HDACi 15a exerts antitumour effects in vitro and in vivo

**Fig. 3:**
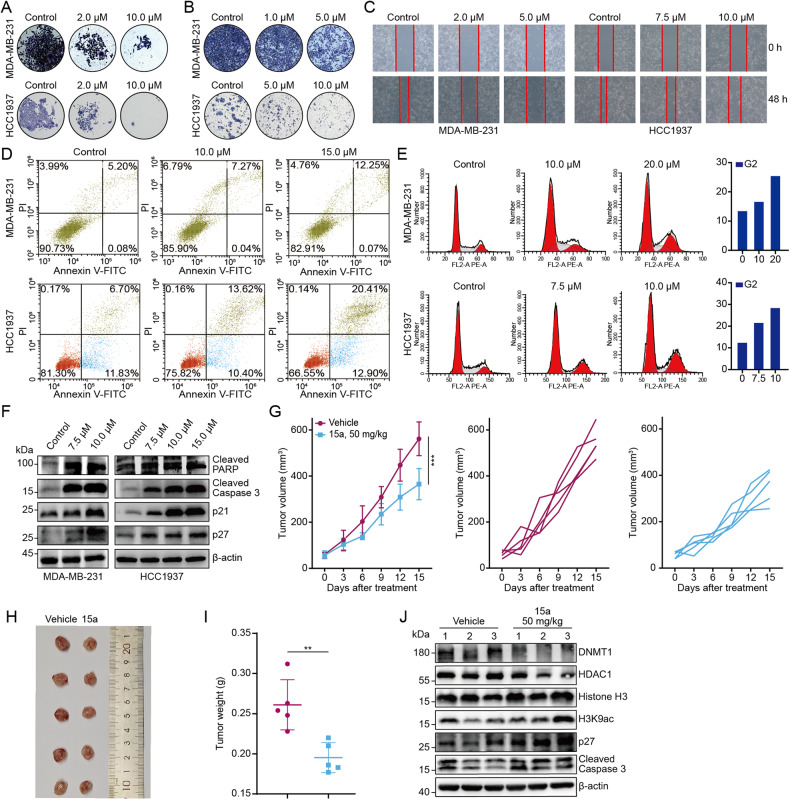
15a inhibits aggressive tumour behaviours and growth in vitro and in vivo. **A** Colony formation assay for MDA-MB-231 and HCC1937 cells. **B** Transwell assays for detecting the migration of MDA-MB-231 and HCC1937 cells. (**C**) Wound healing assay images of MDA-MB-231 and HCC1937 cells after incubation with different concentrations of 15a for 0 h and 48 h. **D** Apoptosis analysis via flow cytometry was performed on MDA-MB-231 and HCC1937 cells treated with different concentrations of 15a for 24 h. **E** Cell cycle analysis via flow cytometry was performed on MDA-MB-231 and HCC1937 cells treated with different concentrations of 15a for 48 h. The percentage of cells in the G2 cycle stage is shown as bar graphs. **F** MDA-MB-231 and HCC1937 cells were treated with different concentrations of 15a for 24 h, and the lysates were collected for immunoblotting with the indicated antibodies. **G** Volumes of 4T1 syngeneic tumours treated with vehicle (*n* = 6) or 15a (*n* = 6). **H** Images of dissected tumours from the vehicle group and 15a treatment group. **I** The weights of 4T1 syngeneic tumours treated with vehicle or 15a are shown as scatter plots. **J** On day 21, tumours harvested from the mice were ground, lysed, and subjected to immunoblotting with the indicated antibodies. **p < 0.01; ***p < 0.001.

A colony formation assay showed that 15a completely inhibited the colony formation of MDA-MB-231 and HCC1937 cells at a certain concentration (Fig. 3A), and the same results were obtained for 4T1 and HCC38 cells (Fig. S1B). We further evaluated the inhibitory effect of 15a on invasion and migration by transwell and wound healing assays, and 15a effectively blocked the invasion of MDA-MB-231 and HCC1937 cells (Fig. 3B). We also found that the migration of MDA-MB-231 and HCC1937 cells was inhibited in a dose-dependent manner after treatment with different concentrations of 15a for 48 h (Fig. 3C). The same results were obtained for HCC38 and 4T1 cells (Fig. S1C, D). We further used a PI-FITC/Annexin assay to evaluate the effects of 15a on apoptosis and the cell cycle and found that 15a induced tumour cell apoptosis [41] and G2 cell cycle arrest in a concentration-dependent manner (Fig. 3D, E). In addition, the expression of apoptosis-related proteins and cell cycle-related proteins in MDA-MB-231 and HCC1937 cells treated with different concentrations of 15a increased to varying degrees (Fig. 3F). The same results were obtained for 4T1, HCC38, MDA-MB-453 and BT-474 cells (Fig. S1E). These results suggest that 15a can inhibit tumour characteristics, promote apoptosis and induce cell cycle arrest.

To further evaluate the antitumour effect of 15a in vivo, 4T1 mouse breast cancer cells were generated from BALB/c wild-type mice. In this study, mice were randomly divided into two groups: those treated with vehicle and those treated with 15a at 50 mg/kg. Compared with the control treatment, 15a gradually suppressed tumour growth, and the tumours in the 15a treatment group were more than 30% smaller in terms of volume than those in the control group (Fig. 3G); however, the difference in tumour weight was not significant (Fig. S1F). And hematoxylin and eosin (HE) staining was conducted on the heart, liver, spleen, lung, and kidney subsequent to euthanizing the mice. The results of the assay substantiated that 15a exhibited no discernible toxicological or adverse impact on the histological composition of the examined normal tissues (Fig. S1G). The tumours in the 15a group were more than 12.5% smaller in terms of weight than those in the control group (Fig. 3H, I). The tumour tissues were homogenized to extract protein, and 15a downregulated the expression of DNMT1 and HDAC1, increased the acetylation of H3, and upregulated the expression of the apoptosis-related protein caspase-3 and the cell cycle-related protein p27 (Fig. 3J). These results suggest that 15a can inhibit the growth of breast cancer tumours in vivo by inhibiting DNMT1 and HDAC1.

### 15a induces a viral mimicry response and activates the ERV signalling pathway by inhibiting the functions of DNMT1 and HDAC1

**Fig. 4:**
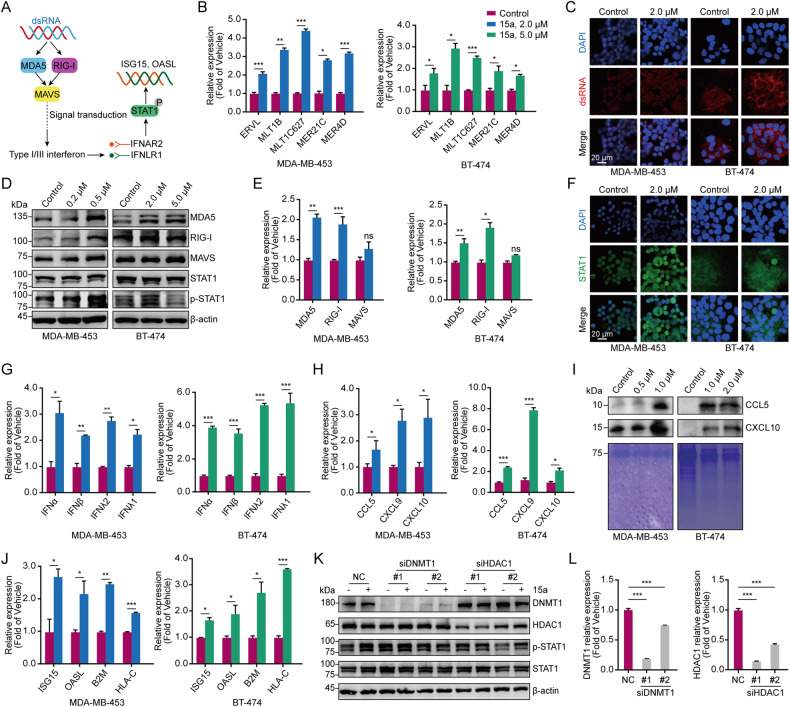
15a induces viral mimicry and activates the IFN signalling pathways. **A** A schematic diagram showing that 15a plays an antitumour role by inducing viral mimicry and endogenous transcription. **B** MDA-MB-453 and BT-474 cells were stimulated with different concentrations of 15a, after which the mRNA levels of ERV-related genes were measured via qRT‒PCR analysis. **C** MDA-MB-453 and BT-474 cells were treated with 15a at 2.0 µM, fixed and subjected to immunofluorescence for dsRNA. **D** MDA-MB-453 and BT-474 cells were treated with different concentrations of 15a, and the lysates were collected for immunoblotting with the indicated antibodies. **E** MDA-MB-453 and BT-474 cells were stimulated with different concentrations of 15a, after which the mRNA levels of ERV signalling pathway-related genes were measured via qRT‒PCR. **F** MDA-MB-453 and BT-474 cells were treated with 15a at 2.0 µM, fixed and subjected to immunofluorescence staining for STAT1. **G** MDA-MB-453 and BT-474 cells were stimulated with different concentrations of 15a, after which the mRNA levels of type І/Ш interferon genes were measured via qRT‒PCR. **H** MDA-MB-453 and BT-474 cells were stimulated with different concentrations of 15a, after which the mRNA levels of chemokine genes were measured via qRT‒PCR analysis. **I** MDA-MB-453 and BT-474 cells were treated with different concentrations of 15a, and the secreted proteins were subjected to immunoblot analysis. **J** MDA-MB-453 and BT-474 cells were stimulated with different concentrations of 15a, after which the mRNA levels of antigen-presenting genes were measured via qRT‒PCR. **K** MDA-MB-453 cells were transfected with 100 nM siRNA against DNMT1 or HDAC1 for 48 h and treated with 15a at 500 nM. Lysates were collected for immunoblotting with the indicated antibodies. (**L**) Verification of the effect of knocking down DNMT1 and HDAC1 in MDA-MB-453 cells. The data points illustrate the mean of three distinct experiments accompanied by standard deviation (SD) values. *p < 0.05; **p < 0.01; ***p < 0.001.

Research has shown that DNMTis increase the intracellular level of dsRNA, resulting in a viral mimicry response, promoting the production of type І and type Ш interferons and inducing the production of a range of ISGs and cytokines [42–44], thereby reversing the immune escape state of tumour cells. There are also published reports that the combination of DNMTis and HDACis has synergistic antitumour effects [45, 46]. Therefore, we conducted a series of experiments to explore whether 15a can induce viral mimicry and promote the production of cytokines and chemokines, thus playing an antitumour role (Fig. 4A). First, after treatment with 15a, the transcript levels of endogenous retrovirus-related genes were increased [37], and the perinuclear accumulation of dsRNA increased in MDA-MB-453 and BT-474 cells (Fig. 4B, C). We then conducted experiments to verify the effect of 15a on the dsRNA signalling pathway. After 15a treatment, key viral RNA-sensing proteins, including MDA5 and RIG-I, were stimulated, and JAK–STAT signalling was activated (Fig. 4D, E). The same results were obtained for 4T1 and HCC38 cells (Fig. S2A, B). We also found that the perinuclear accumulation of STAT1 increased in MDA-MB-453 and BT-474 cells (Fig. 4F). We speculated that 15a treatment restored type І/Ш IFN signalling in MDA-MB-453 and BT-474 cells, as indicated by the increase in STAT1 phosphorylation (Fig. 4D). The results also confirmed that the transcript levels of type І/Ш interferons in MDA-MB-453 and BT-474 cells treated with different concentrations of 15a were increased compared with those in untreated cells (Fig. 4G). The same results were obtained for 4T1 and HCC38 cells (Fig. S2C). In addition, the transcription of antigen presentation-related genes, including B2M and HLA-C; the transcription of ISGs, including ISG15 and OASL, which are involved in interferon response activation (Fig. 4J); and the transcription and secretion of the chemokines CCL5 and CXCL10 were promoted after 15a treatment in MDA-MB-453 and BT-474 cells (Fig. 4H, I). The results indicated that the expression levels of DNMT1 and HDAC1 were significantly reduced; knocking down the demethylase DNMT1 induced highly similar results, but knocking down the deacetylase HDAC1 had little effect (Fig. 4K, L). Overall, 15a induces the interferon response, promotes antigen presentation and induces chemokine production by inducing viral mimicry and activating the ERV signalling pathway.

### Depletion of pattern recognition receptors and interferon receptors abrogates 15a-induced activation of the IFN signalling pathway

**Fig. 5:**
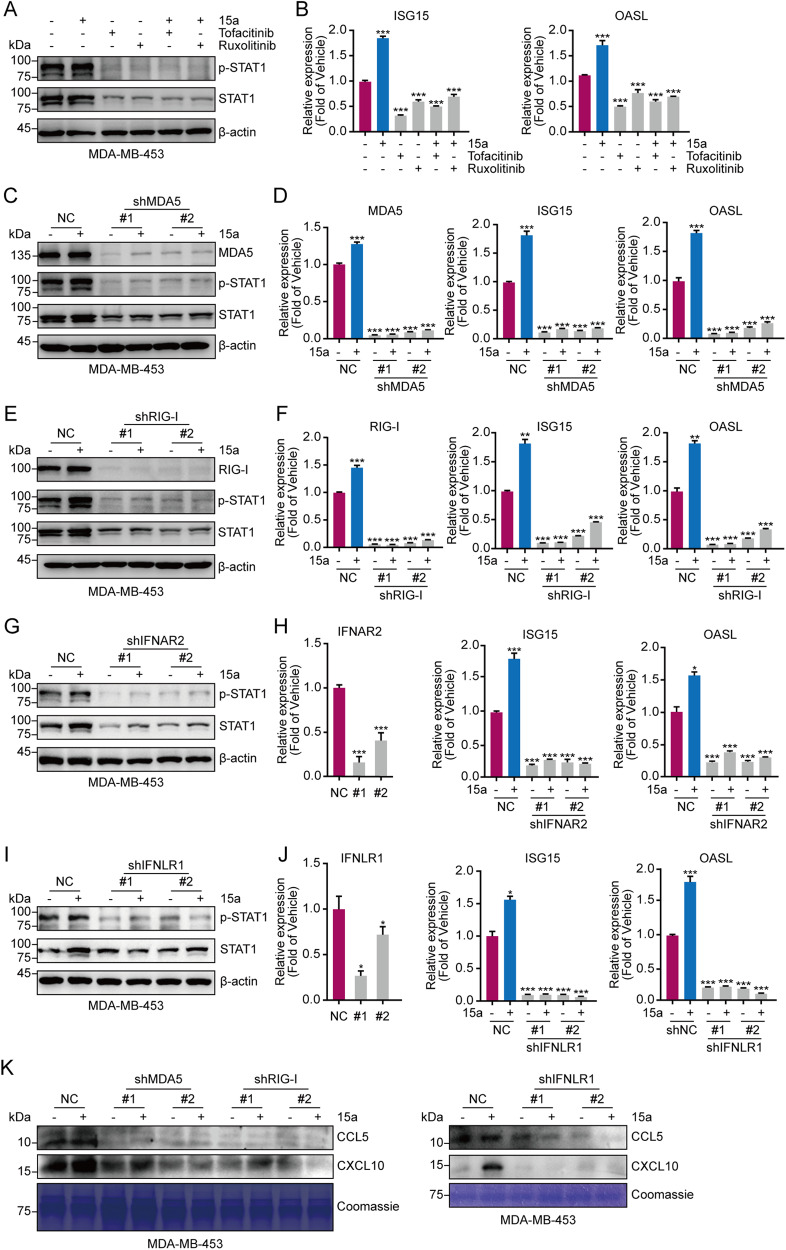
15a restores IFN signalling via the RIG-I–MAVS pathway. **A** MDA-MB-453 cells were treated with 15a at 500 nM, tofacitinib at 1.0 µM and ruxolitinib at 1.0 µM for 48 h, after which the lysates were collected for immunoblotting with the indicated antibodies. **B** MDA-MB-453 cells were treated with 15a, tofacitinib or ruxolitinib for 48 h, after which the mRNA levels of ISGs were measured via qRT‒PCR analysis. **C**‒**K** MDA-MB-453 cells were transduced with MDA5, RIG-I, IFNAR2, IFNLR1-specific shRNA (#1, #2), or NC. (C/E/G/I) Whole-cell lysates extracted from transduced and 15a-treated cells were subjected to immunoblot analysis with the indicated antibodies. **D**/**F**/**H**/**J**) Total RNA was extracted from the transduced cells. qRT‒PCR analysis of MDA5, RIG-I, IFNAR2, IFNLR1, ISG15, and OASL was performed. The values represent the amount of mRNA relative to the control. (**K**) MDA-MB-453 cells were transduced and treated with 15a for 48 h, after which the secreted proteins were subjected to immunoblot analysis. *p < 0.05; **p < 0.01; ***p < 0.001.

We speculated that the increase in ISG expression after 15a treatment might be caused by an increase in the production of IFN in tumour cells. JAKs mediate intracellular signalling after ligand-dependent activation of IFN receptors, and the JAK inhibitors tofacitinib and ruxolitinib completely mitigated 15a-induced increases in p-STAT1 and total STAT1 and reversed the transcription of ISG15 and OASL activated by 15a in MDA-MB-453 cells (Fig. 5A, B). The same results were obtained for 4T1 and HCC38 cells (Fig. S2D). These results suggest that 15a can induce tumour cell production of IFN, which in turn activates the JAK–STAT pathway. Furthermore, after knocking down the dsRNA key sensors MDA5 and RIG-I, the increases in p-STAT1 and total STAT1 expression were abrogated in the MDA-MB-453 cell line (Fig. 5C, E). Moreover, the secretion of the chemokines CCL5 and CXCL10 was reduced (Fig. 5K). Moreover, depletion of MDA5 and RIG-I by shRNA abrogated the 15a-induced increase in the expression of ISG15 and OASL (Fig. 5D, F). Compound 15a did not further enhance IFN signalling in MDA5 or RIG-I knockout cells, further suggesting that 15a-mediated IFN gene activation requires the MDA5 and RIG-I pathways. Notably, knockdown of MAVS abrogated 15a-induced p-STAT1 and total STAT1 expression in 4T1 and HCC38 cells (Fig. S2E). Like for the key sensors MDA5 and RIG-I, knocking down the type І IFN receptor IFNAR2 and the type Ш IFN receptor IFNLR1 also abrogated the 15a-induced increase in p-STAT1 and total STAT1 expression (Fig. 5G, I and Fig. S2F, H, J), and the same effects were also observed for ISG15 and OASL (Fig. 5H, J and Fig. S2G, I, K). Subsequently, depletion of MDA5, RIG-I, and IFNLR1 abrogated the 15a-induced secretion of CCL5 and CXCL10 (Fig. 5K). We thus concluded that 15a triggers virus sensors to induce an interferon response.

### 15a sensitizes tumours to anti-PD-L1 immunotherapy in breast cancer models

**Fig. 6:**
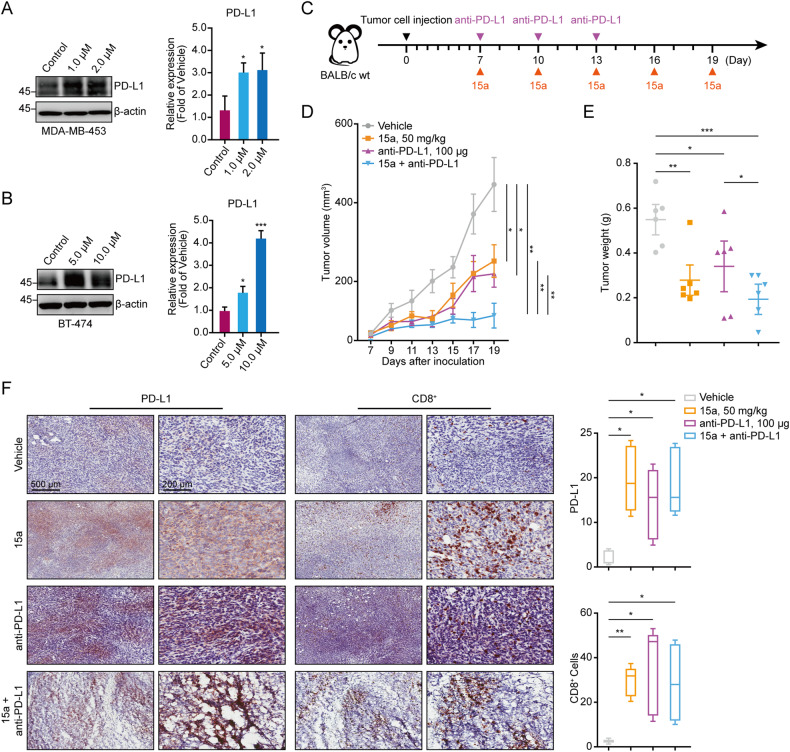
The combination of 15a and an anti-PD-L1 antibody suppressed tumour growth in vivo. **A**, **B** MDA-MB-453 and BT-474 cells were stimulated with different concentrations of 15a for 48 h, after which western blot and qRT‒PCR analyses were performed to measure the levels of PD-L1. **C** Schematic outline of 15a and anti-PD-L1 treatment for tumours. **D** Volumes of 4T1 syngeneic tumours treated with vehicle, 15a, anti-PD-L1, or the combination of 15a and anti-PD-L1 (*n* = 6). **E** The weights of 4T1 syngeneic tumours treated with vehicle, 15a, anti-PD-L1, or the combination of 15a and anti-PD-L1 are shown as scatter plots. **F** Representative images of PD-L1 and CD8^+^ T-cell staining in 4T1 syngeneic tumours subjected to different treatments, including vehicle, 15a, anti-PD-L1, or the combination of 15a and anti-PD-L1, were obtained. IHC was performed on tumour sections, which were then scanned and analysed at a magnification of 20× using ImageJ software. *p < 0.05; **p < 0.01; ***p < 0.001.

In general, PD-L1 is widely recognized as a dependable biomarker for ICB, and patients with negative PD-L1 expression appear to experience less favourable outcomes from ICB than patients with positive PD-L1 expression. Given that PD-L1 expression is associated with downstream IFN signalling [47–49], we also validated that 15a significantly enhances PD-L1 expression in breast cancer cells at both the protein and mRNA levels (Fig. 6A, B and Fig. S3A). Consequently, we conducted an in vivo experiment to investigate whether 15a can effectively sensitize tumours to immunotherapy by establishing orthotopic 4T1 tumours in immunocompetent BALB/c mice. When the tumours reached an average volume of 100–200 mm^3^, the mice were randomly divided into four groups and received treatment with either vehicle or 15a along with an anti-PD-L1 antibody (Fig. 6C). The administration of 15a and anti-PD-L1 monotherapy resulted in a significant delay in tumour growth compared to that of the control treatment. Furthermore, the combination of 15a and anti-PD-L1 therapy significantly inhibited tumour growth compared to that in both the control treatment and monotherapy groups (Fig. 6D, E and Fig. S3B). Throughout the treatment period, the body weights of the mice in all four groups remained relatively stable, indicating the safety of the combination treatment (Fig. S3C). The IHC data revealed greater PD-L1 and CD8 staining in the treatment group than in the vehicle group (Fig. 6F). This finding suggested an increase in immune infiltration within the tumour microenvironment. Collectively, these findings provide evidence that targeting DNMT1 and HDAC1 enhances the expression of PD-L1, and the concurrent administration of an anti-PD-L1 agent represents a novel approach for the treatment of breast cancer.

## Discussion

Breast cancer is a common cancer worldwide, and conventional treatment includes surgical removal of malignant tissue, ionizing radiation, and chemotherapy, all of which can cause a variety of side effects. Moreover, cancer resistance and recurrence are pressing concerns with conventional cancer treatment. Therefore, new treatment options for breast cancer are needed. The field of epigenetic therapies is gaining recognition as a promising area of study for various types of tumours [50, 51]. Many studies have indicated that the associations between breast cancer development and resistance to therapy are linked to epigenetic nonmutations, including aberrant DNA methylation and histone modifications. While there are some currently approved HDAC inhibitors and hypomethylating agents, it is important to note that posttranscriptional gene regulation often involves multiple levels of control. This finding implies that a combination of drugs targeting different epigenetic mechanisms may be a more effective therapeutic approach than single-drug treatment [35]. This study provides evidence of the feasibility of the use of a single compound that effectively targets DNMTs and HDACs, demonstrating its substantial efficacy in vitro and in vivo against various forms of breast cancer characterized by an unfavourable prognosis.

The results suggested that 15a is a versatile antitumour agent that has multiple antitumour biological effects. First, 15a induces apoptosis and cell cycle arrest in human breast cancer cells. Additionally, it effectively inhibits the migration and invasion of aggressive breast cancer cells. Furthermore, 15a significantly suppressed the proliferation of 4T1 tumour cells and notably suppressed in tumour growth in mouse breast cancer models. Taken together, these results indicate that 15a exerts multiple antitumour effects by concurrently inhibiting DNMTs and HDACs.

Recent studies suggest that inhibitors of DNMTs and HDACs induce antitumour effects by stimulating viral mimicry responses, stimulating type І and Ш interferon reactions, and initiating a sequence of cascade signalling reactions [52, 53]. Furthermore, our investigation confirmed that 15a may also induce antitumour effects via this particular mechanism of action. In preclinical models, DNMTis and HDACis were shown to restore cytotoxic T-cell functions and reverse immunosuppressive effects of the tumour microenvironment; they were also found to exert synergistic effects with ICB, supporting clinical trials [52]. Recent studies have shown the contribution of type І interferon responses to the effectiveness of checkpoint inhibitors, and the use of 15a in combination with immune checkpoint inhibitors such as anti-PD-L1 agents could serve as a compelling therapeutic approach [30, 54].

In conclusion, 15a has significant potential as a novel and effective dual inhibitor of DNMTs and HDACs and increased the survival rate in in vivo models of breast cancer. This effect is attributed, at least in part, to the induction of a viral mimicry response. Consequently, 15a represents a promising drug candidate for the safe and efficient targeting of cancer, thereby opening avenues for the treatment of various human tumours with unfavourable prognoses.

## Materials and methods

### Cell culture and reagent

MDA-MB-453, MDA-MB-231, MDA-MB-468, MDA-MB-436, HCC1937, BT-474, T-47D, MCF-7, BT-549 and 4T1 cells were generously provided by the Cell Bank, Chinese Academy of Sciences. MDA-MB-361 cells were acquired from Procell. MDA-MB-157, HCC38, and HCC1143 cells were obtained from Nanjing Kebai Biological. T-47D, MCF-7, 4T1, MDA-MB-157, and BT-549 cells were cultured in Dulbecco’s modified Eagle’s medium (DMEM) supplemented with 10% fetal bovine serum (FBS) and 1% penicillin/streptomycin. HCC1937 cells were cultured in Roswell Park Memorial Institute (RPMI) 1640 medium supplemented with 10% FBS, 1% penicillin/streptomycin, and 100 μM sodium pyruvate. The BT-474, HCC38, and HCC1143 cell lines were cultured in RPMI 1640 medium supplemented with 10% FBS and 1% penicillin/streptomycin. The MDA-MB-453, MDA-MB-231, MDA-MB-468, and MDA-MB-436 cell lines were cultured in Leibovitz’s L-15 medium supplemented with 10% FBS and 1% penicillin/streptomycin. The MDA-MB-361 cell line was cultured in Leibovitz’s L-15 medium supplemented with 20% FBS and 1% penicillin/streptomycin. The MDA-MB-453, MDA-MB-231, MDA-MB-468, MDA-MB-436, and MDA-MB-361 cell lines were incubated at 37 °C in a humid atmosphere without CO_2_, while the remaining cell lines were incubated at 37 °C in a humid atmosphere with 5% CO_2_.

### Cell viability assay

MTT assay was performed on breast cancer cells currently available in the laboratory to evaluate the antiproliferative activity of the compound. DNMT inhibitor (SGI-1027) and HDAC inhibitor (SAHA) were used as positive control compounds. Different breast cancer cell density is different, the range is 2× 10^6^–1 × 10^7^, with different concentrations of 15a treatment. After 72 h treatment, 10 µL MTT solution (5 mg/mL in 1× PBS) was added to each well. The formaldehyde crystals were dissolved in 100 µL DMSO per well after incubation at 37 °C for 4 h. The absorbance at 490 nm was measured using SpectraMax i3x. Three independent experiments were conducted.

### Combination assay

The combination index (CI) values were calculated by determining the growth inhibition of MDA-MB-453 and BT-474 cells at various concentrations of SGI-1027 (0.1, 0.2, 0.5 and 1.0 µM) in conjunction with multiple concentrations of SAHA (0.1, 0.2, 0.5 and 1.0 µM). The obtained data were analyzed using the methodology outlined in CompuSyn software. The CI values were utilized to ascertain whether the drug combinations exhibited synergistic, additive, or antagonistic effects. The concepts of synergy, additivity and antagonism were operationalized using a CI value less than one, equal to one, and greater than one, respectively. The selection of the combination study design was predicated on the IC_50_ values of the assayed molecules, namely SGI-1027 and SAHA, against the tested cell line. In both instances, the IC_50_ values were found to be less than 5.0 µM.

### Immunoblotting

The total protein was extracted by lysing the cells in ice-cold Nonidet P-40 buffer containing 1× protease inhibitor and 1× phosphatase inhibitor. The cells were then scraped off using cell scraping and subjected to 5 s ultrasound using a scientz-iiD ultrasonic crusher. Following centrifugation at 14,000 × *g* for 10 min, the supernatant was collected for determination of protein concentration using the BCA assay. The remaining supernatant was mixed with 5× loading buffer (including 5 M DTT) in a volume ratio of 1/5 and heated in a metal bath at 95 °C for 10 min.

Secretory proteins were extracted by culturing breast cancer in a 6 cm petri dish for 12 h and allowing it to attach to the wall. The old medium was then removed and the cells were washed three times with 1× PBS before being transferred to serum-free medium. Following treatment with varying concentrations of 15a for 48 h, the supernatant was collected by centrifuging at 1000× *g* for 5 min to eliminate cell debris. A mixture of 0.5 mL supernatant medium, 0.5 mL methanol, and 0.125 mL chloroform was then centrifuged at 13,000× *g* for 5 min. The upper phase was discarded, and an additional 0.5 mL of methanol was introduced. Subsequently, the samples underwent centrifugation at a speed of 13,000× *g* for a duration of 5 min. The resulting supernatants were discarded, and the pellets were subjected to drying at a temperature of 50 °C for a period of 5 min. Loading buffer was then added, and the samples were subsequently boiled for a duration of 10 min. Electrophoresis of lysed proteins was carried out using a commercially available gel (ExCell) at varying concentrations, followed by transfer onto a PVDF membrane (Merck). The membranes were blocked in a solution of 1× TBS containing 5% nonfat dry milk and 0.1% Tween 20 for a duration of 1 h, and subsequently incubated with a specific primary antibody at a temperature of 4 °C overnight. Bound antibodies were detected with horseradish peroxidase (HRP) secondary antibodies (Beyotime) and visualized by enhanced chemiluminescence reagent (Bio-Rad). Catalog numbers of primary antibodies are listed in Supplementary Table S1.

### Flow cytometry analysis

MDA-MB-231 and HCC1937 were treated with either vehicle or 15a for 24 or 48 h. Using Cell Cycle and Apoptosis Kit (Yesen) and Annexin V-FITC/PI Apoptosis Detection Kit (Beyotime), the samples were made according to the instructions. CytoFLEX flow cytometry was used to detect the number of cells in different cycles and the level of apoptosis.

### RNA extraction and quantitative real-time PCR assay (qRT‒PCR)

According to the instructions, total RNA was extracted from the cells using the Eastep Super total RNA extraction kit, and the extracted total RNA was quantifiable by Nanodrop spectrophotometer with acceptable purity. For qRT‒PCR, the total RNA was reverse-transcribed by Bio-Rad PCR after different volume samples and 4 μL Hifair^®^ III 1st Strand cDNA Synthesis SuperMix reagent were added according to the calculation (reversing 1 μg of RNA). Using 2 μL cDNA, 10 μL Hieff^®^ qPCR SYBR Green Master Mix (Low Rox Plus), and 0.5 μL forward and reverse primers, using 7500 Fast system under 20 μL volume reaction condition, qPCR was performed using 20 μg cDNA at 95 °C, 5 min, 95 °C, 10 s 60 °C 30 s 40 cycles. The primers used in this study are listed in the Supplementary Table S2.

### Transwell evaluation

The 8 μm pore membrane chamber (Corning) was placed in a 24-well plate, and the cells with a density of 4 × 10^5^ were placed in the chamber. The 24-well plate was added with 1 mL medium containing either vehicle or different concentrations of 15a, which was treated for 48 h. After the chamber was washed three times with 1× PBS, adding 1 mL Bourn’s Tissue fixative into the chamber and fixing for 1 h. Then washing three times with 1× PBS after fixing, add 1 mL crystal violet solution dyeing solution for 1 h, and finally wash three times with 1× PBS, wipe the chamber gently with a cotton swab, and then take images with an inverted fluorescence microscope after drying. Three fields of view were taken from each sample.

### Colony-forming assay

MDA-MB-231 and HCC1937 cells were seeded in 6-well plates at a density of 300 cells/mL and treated with either vehicle or 15a for 14–21 d, replacing the medium with fresh medium every 72 h. Subsequently, the formed colonies were fixed with 1 mL Bourn’s Tissue fixative for 1 h, washed three times with 1× PBS after fixation, added 1 mL crystal violet solution staining solution for 1 h, and finally washed three times with 1× PBS, dried and then photographed with an inverted fluorescence microscope. Three fields of view were taken for each sample.

### Wound healing assay

MDA-MB-231 and HCC1937 cells were cultured in 6-well plates at a density of 2 × 10^5^ cells/mL. Upon reaching confluence, the cells were subjected to serum starvation using FBS-free medium for a duration of 12 h. Prior to cell seeding, three parallel lines were marked on the bottom of the 6-well plates. Subsequently, wounds were induced by vertically scratching the cell monolayer with 200 µL sterile pipette tips along the aforementioned lines. Following a thorough washing and medium replacement, the cells were exposed to either a control solution or varying concentrations of 15a. The wounds adjacent to the lines were captured through photography at the initial time point and 48 h following the act of scratching, employing an inverted fluorescence microscope equipped with a 10× objective lens. For each sample, three distinct fields of view were documented.

### Thermal shift assay

The cells resuspended in 1× protease inhibitor was subjected to freezing using liquid nitrogen and subsequent thawing at 37 °C in a water bath. Once approximately 60% of the cells were thawed, they were transferred to ice to continue thawing, repeating this process three times. The soluble proteins in the supernatant were separated from the cell precipitation by centrifugation at 20,000× *g* for 20 min at 4 °C. The resulting supernatants were then treated with both a vehicle and 15a. Following a 5 min incubation at room temperature with either a vehicle or drugs, the lysates were partitioned into multiple 50 µL aliquots within new 200 µL PCR tubes. These aliquots were then individually heated at varying temperatures for 3 min using a thermal cycler, followed by a 3 min cooling period at room temperature. Subsequently, the heated lysates underwent centrifugation at 20,000× *g* for 20 min at 4 °C to separate soluble proteins from precipitated proteins. The resulting soluble proteins were utilized for western blotting, with their protein concentration determined through the employment of a BCA assay.

### Transient transfection cell assay

Cells were initially plated at a confluency range of 50% to 60% in 60 mm plates and incubated for a duration of 24 h. Subsequently, the cells were transiently transfected using Lipofectamine RNAiMAX Transfection Reagent, employing two sets of pooled siRNAs per gene, specifically DNMT1, HDAC1, MDA5, RIG-I and MAVS, which were procured from GenePharma. To serve as a control, nonspecific scrambled siRNA was utilized. Following a 24-hour period of transfection, the cells were lysed for the purpose of immunoblotting analysis. The gene sequence can be found in Supplementary Table S3.

### Construction of stable cells

Extraction of plasmid: take a conical bottle and pour 100 mL LB medium, seal with tissue culture sealing film and rubber band, and then sterilize with high-pressure steam. After cooling to room temperature, ampicillin was added (final concentration was 100 μg/mL), 500 μL of sequenced bacterial solution was added, and the bed was shaken at 37 °C at 200× *g* overnight. After the bacterial solution was cloudy, the plasmid was extracted in large quantities (Beyotime), and the gene sequence was shown in Supplementary Table S4. (2) Packaged virus: HEK-293T cells were incubated in DMEM medium at 37 °C. The mixture (4 μg target plasmid, 1 μg pMD2.G envelope plasmid, 3 μg psPAX2 package plasmid and 10 μL (1 mg/mL) PEI (Beyotime, C0537) was added when the density of cells was 80%–90%. The mixture was prepared with FBS-free medium to 400 μL and then left for 10 min. Drips were added to 293 T cells in 10 cm petri dish. After 6–8 h, the medium was discarded and was added into 5 mL DMEM medium. After 24 h, the venom was collected and was centrifuged for 1000× *g* for 5 min, and the supernatant was divided into 1 mL tubes and stored at −80 °C. (3) Infected cells: the target cells were spread in a 6 cm dish and incubated overnight at 37 °C. When the cell density was 80–90%, the old medium was discarded and 1 mL of disease venom and 1 μL polybrene (8 mg/mL, Beyotime, C0351) were added. After 4 h, the disease venom was sucked away and added to 3 mL of complete medium, or directly added to 2 mL of complete medium. In order to screen out the successfully infected cells, complete medium containing puromycin was added to the next passage and subsequent culture for culture, and after the state of the target cells was stable, western blot assay and qRT‒PCR assay were used to verify whether the knockdown was carried out.

### Immunofluorescence (IF)

Breast cancer cells with a density of 50–60% were cultured in confocal petri dish (Nest, KH-TB-A1111) for 48 h, then fixed with −20 °C pre-cooled ice methanol at room temperature for 10 min, and washed three times with 1× PBS after fixation. Goat serum blocking buffer (1× PBS/5% normal serum / 0.3% Triton X-100) was added, enclosed at room temperature for 1 h, washed three times with 1× PBS, STAT1 (1:200) and J2 (1:200) antibody, incubated overnight at 4 °C, recovered primary antibody on the second day, washed three times with 1× PBS, and added rabbit secondary antibody (1:200) or mouse secondary antibody (1:50), after incubation at room temperature for 1 h, wash three times with 1× PBS, add DAPI anti-fluorescence quencher (Yesen, 36308ES20), and incubate in dark light for 5 min before taking photos. The primary and secondary antibodies were formulated with a dilute release buffer (1× PBS / 1% BSA / 0.3% Triton X-100).

### Methylation-specific PCR (MSP) analysis

Cells were subjected to treatment with a vehicle and different concentrations of 15a. The Blood/Cell/Tissue Genomic DNA Extraction Kit (Tangen) was used to extract DNA from these cells. Subsequently, the DNA was processed using the D5005/EZDNAMethylation-GoldKit kit (Zymo Research) and the methylation-specific PCR kit (Tangen). During this process, unmethylated cytosine was converted to uracil, while methylated cytosine remained unaltered. Based on this alteration in the DNA bases, primers targeting methylated and unmethylated sequences were designed and amplified using PCR.

### In vivo functional assays

A total of 2 × 10^5^ 4T1 cells were subcutaneously inoculated into female BALB/c wild-type mice aged 6–8 weeks, which were obtained from Guangdong Pharmaceutical Company. Once the tumour volume reached 80–100 mm^3^, the mice were randomly assigned to two groups, with five mice in each group. The groups were administered either a vehicle (10% DMSO, 45% PEG300, and 45% 1× PBS) or 15a (dissolved in vehicle) intravenously once daily. Tumour size was measured using vernier calipers every three days. The mean tumour volume for each group was calculated using the formula (length × width × width)/2 and expressed in cubic millimeters. The animals were killed on day 21, and the removed tumour tissue was weighed and photographed. For in vivo combination therapy, mice were treated with vehicle or 15a alone injected on once every three days or in combination with anti-PD-L1 antibody (BioXcell, 100 µg injected on days 7, 10, 13). The experiments were conducted in accordance with the regulations of the Agency’s Animal Management and Use Committee and were approved by the Committee.

### Hematoxylin and eosin (HE) staining

The organ specimens, comprising the heart, liver, spleen, lung, and kidney, were subjected to a series of procedural steps, followed by staining with the HE staining kit (Biosharp).

### Immunohistochemistry (IHC)

The tumour specimen underwent a series of procedures, including dewaxing and washing with 1× PBS, treatment with 3% H_2_O_2_ for 20 min to prevent endogenous peroxidase activity, heat-induced epitope retrieval at EDTA (pH 9.0) for 2 min, and incubation in a blocking buffer containing 10% goat serum for 30 min at room temperature. Subsequently, the sections were exposed to primary antibodies against PD-L1 (CST, 64988, 1:200) and CD8α (CST, 98941, 1:200) overnight at 4 °C, followed by incubation with secondary antibodies (MaxVision) for 1 h at room temperature. Finally, the sections were developed using diaminobenzidine (DAB). The IHC profiler plugin in Image J software was utilized to automatically analyze the percentage and staining intensity of positive cells in each image [55, 56].

### Database analysis

Gene correlation analysis was retrieved from the TCGA database (https://portal.gdc.cancer.gov/) RNA expression profiles and matched clinicopathological information of breast cancer patients. Spearman correlation analysis was conducted on DNMT1 and HDAC1 in tumor and non-tumor groups using R4.2.1 to explore the relationship between DNMT1 and HDAC1. A statistically significant difference was defined as a difference with a *p*-value < 0.05. Kaplan–Meier overall survival curves of human breast tumors generated using Kaplan–Meier Plotter according to DNMT1 and HDAC1 gene expression levels. The R package ggplot2 was used to plot gene difference analysis, correlation analysis and Kaplan–Meier survival curves.

### Statistical analysis

All experiments requiring statistical analysis were independently conducted with a minimum of three replications. The experimental data are presented as the mean value ± standard deviation (SD) or standard error of the mean (SEM). Statistical analysis was carried out using GraphPad Prism 8.0. Differences were assessed an unpaired Student’s *t* test for two-sample comparisons and one-way analysis of variance (ANOVA) for multiple-sample comparisons. A statistically significant difference was defined as a *p*-value < 0.05. Spearman correlation analysis was employed for assessing correlations between DNMT1 and HDAC1 expression levels. Kaplan–Meier survival curves were analyzed using the Mantel–Cox log-rank test. Inclusion/exclusion criteria were all pre-established and no samples or animals were excluded from the analysis.

### Supplementary information

Supporting Information

Original Data File

aj-checklist

## Data Availability

The data that support the findings of this study are available from the corresponding author upon request.
