# Supplementary material for: Dual inhibitors of DNMT and HDAC induce viral mimicry to induce antitumour immunity in breast cancer
Source: Cell Death Discov. 2024 Mar 15;10:143. doi: 10.1038/s41420-024-01895-7 (PMC10943227; doi:10.1038/s41420-024-01895-7)
Supplement: Supplementary file 1 — Supporting Information [file 41420_2024_1895_MOESM1_ESM.docx]

# Supporting Information

**Dual inhibitors of DNMT and HDAC induce viral mimicry to induce antitumour immunity in breast cancer**

**Figs. S1 to S3**


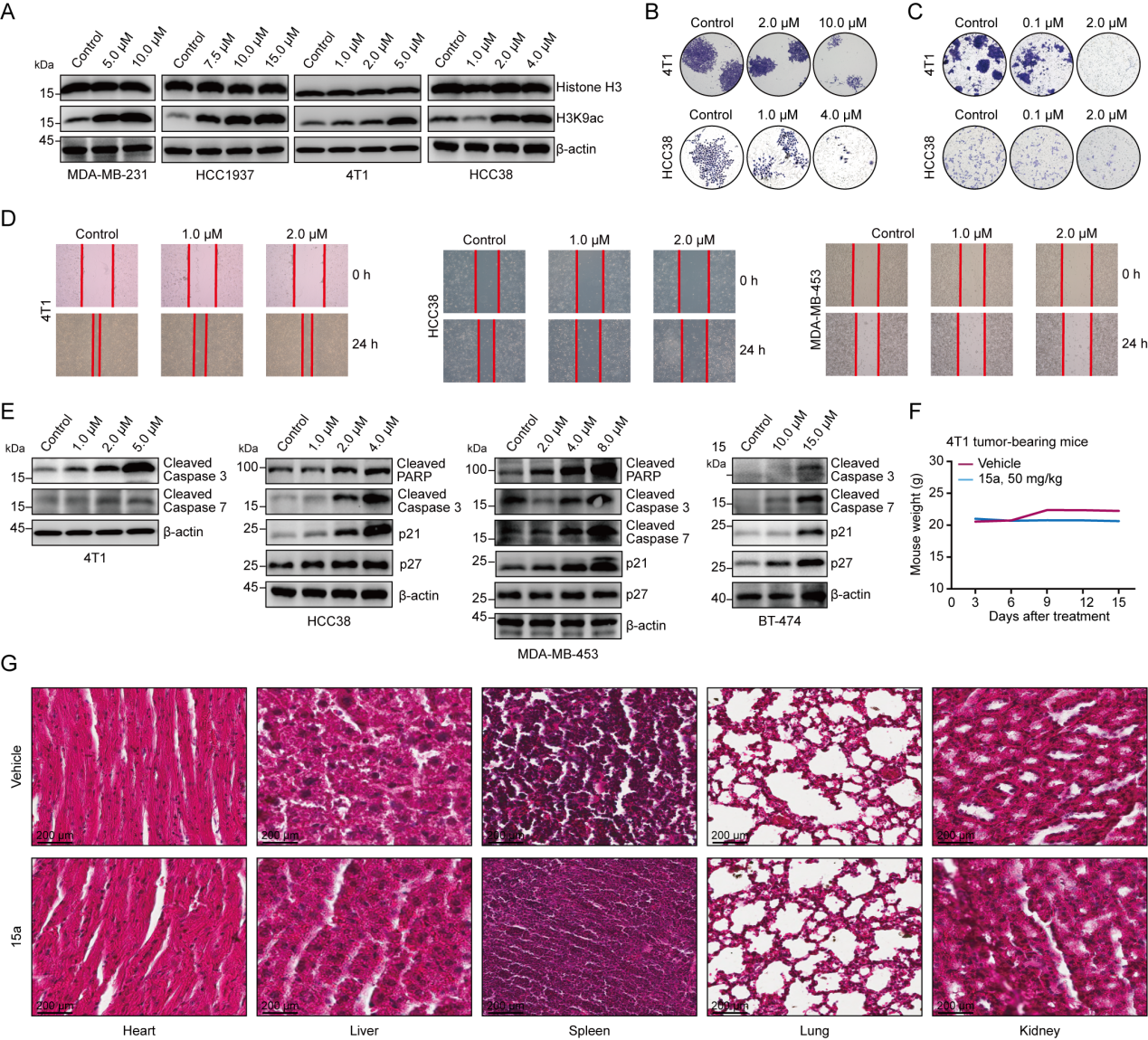


**Figure S1.** (A) MDA-MB-231, HCC1937, 4T1, and HCC38 cells were treated with different concentrations of 15a for 24 h, and the lysates were collected for immunoblotting with the indicated antibodies. Please note that the β-actin of HCC1937 cell shown here is the same that is shown for Figure 3F, because they were both derived from the same blot. (B) Colony formation assay for 4T1 and HCC38 cells. (C) Transwell assay for detecting cell migration ability in 4T1 and HCC38 cells after incubation with different concentrations of 15a. (D) Wound scratch healing assay images of 4T1, HCC38, and MDA-MB-453 cells after incubation with different concentrations of 15a for 0 h and 24 h. (E) 4T1, HCC38, MDA-MB-453, and BT-474 cells were treated with different concentrations of 15a for 24 h, and the lysates were collected for immunoblotting with the indicated antibodies. Please note that the β-actin of 4T1, HCC38, and BT-474 cells cell shown here is the same that is shown for Figure 2A and Figure S1A, because they were both derived from the same blot. (F) Mouse weights of BALB/c wild-type mice injected with 4T1 cells treated with the indicated drugs are shown. (G) Representative images of the heart, liver, spleen, lung, and kidney staining in BALB/c wild-type mice injected with 4T1 cells subjected to different treatments, including vehicle and 15a, were obtained. H&E was performed on organ sections, which were then scanned at a magnification of 20×.


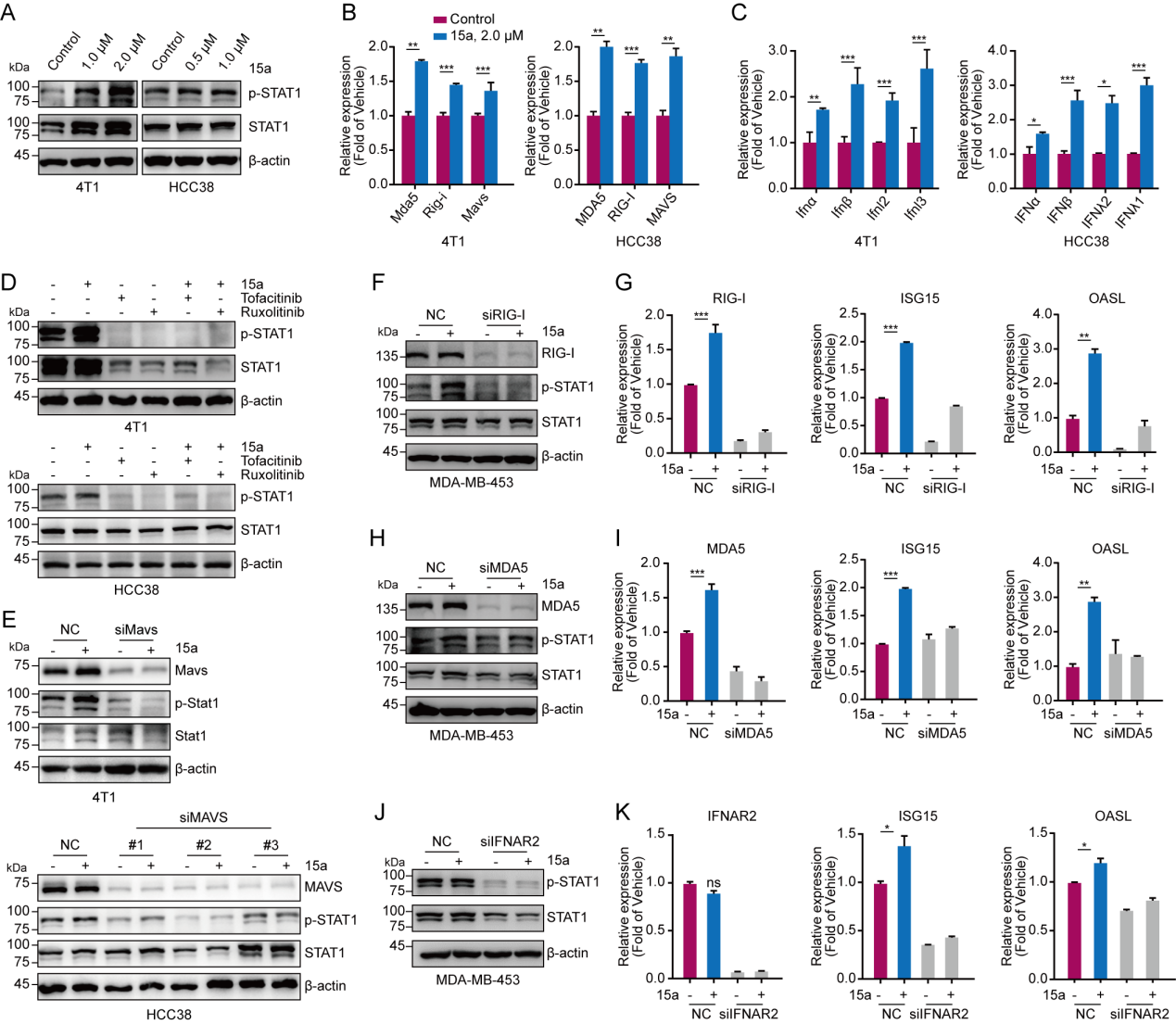


**Figure S2.** (A) 4T1 and HCC38 cells were treated with different concentrations of 15a for 48 h, and the lysates were collected for immunoblotting with the indicated antibodies. (B) 4T1 and HCC38 cells were stimulated with different concentrations of 15a for 24 h, after which the mRNA levels of ERV signalling pathway-related genes were measured via qRT‒PCR. (C) 4T1 and HCC38 cells were stimulated with different concentrations of 15a for 24 h, after which the mRNA levels of type І/Ш interferon genes were measured via qRT‒PCR analysis. (D) 4T1 and HCC38 cells were treated with 15a (2.0 µM), tofacitinib (1.0 µM), or ruxolitinib (1.0 µM) for 48 h, after which the lysates were collected for immunoblotting with the indicated antibodies. (E) 4T1 and HCC38 cells were transfected with 100 nM siRNA against MAVS for 48 h, reseeded and treated with 15a (2.0 µM). Lysates were collected for immunoblotting with the indicated antibodies. (F–K) MDA-MB-453 cells were transfected with 100 nM siRNA against MDA5, RIG-I or IFNAR2 for 48 h, reseeded and treated with 15a (2.0 µM). (F/H/J) Whole-cell lysates extracted from the transfected and 15a-treated cells were subjected to immunoblot analysis with the indicated antibodies. (G/I/K) Total RNA was extracted from the transfected cells. qRT‒PCR analysis of MDA5, RIG-I, IFNAR2, ISG15, and OASL was performed. The values represent the mRNA levels relative to those in the NC group. * p < 0.05; ** p < 0.01; *** p < 0.001.


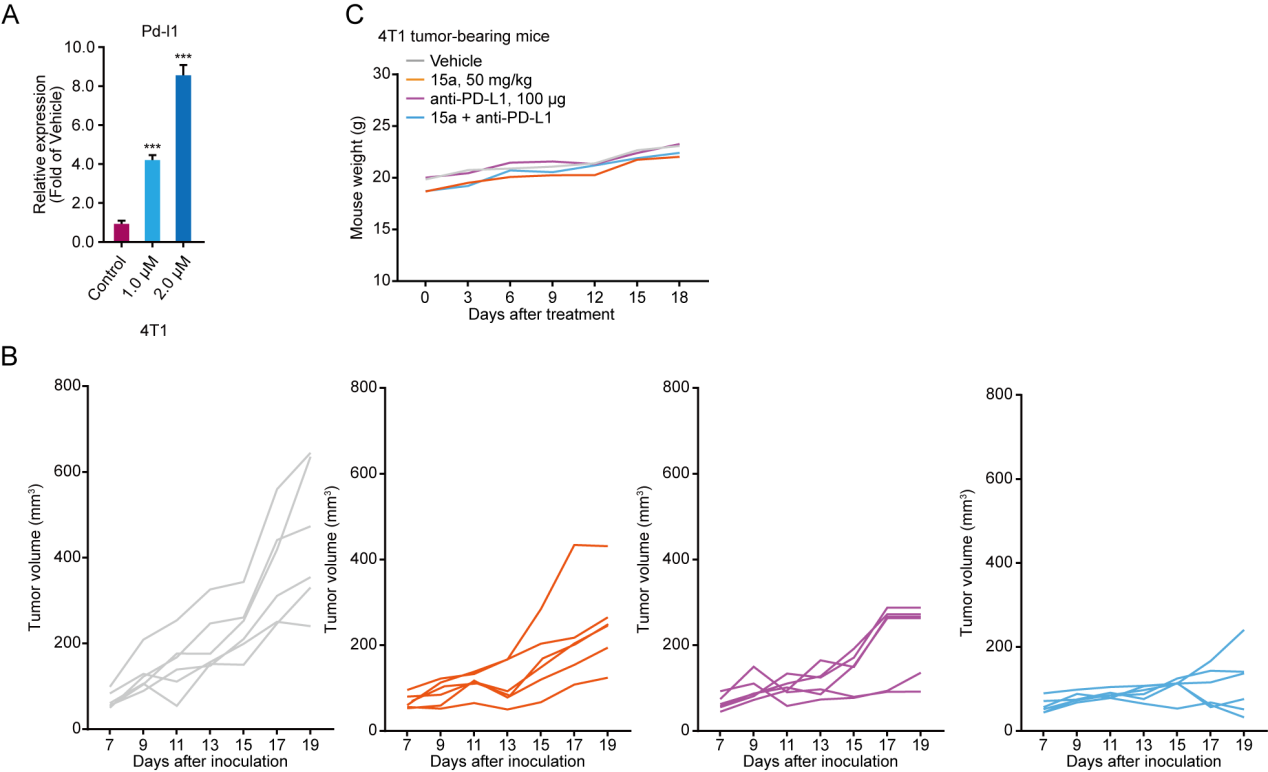


**Figure S3.** (A) Mouse weights of BALB/c wild-type mice injected with 4T1 cells and treated with different drugs are shown (after intraperitoneal injection daily; six mice per group). (B) 4T1 cells were stimulated with different concentrations of 15a for 24 h, after which the mRNA levels of Pd-l1-related genes were measured via qRT‒PCR analysis. (C) Volumes of 4T1 syngeneic tumours treated with vehicle, 15a, anti-PD-L1, or the combination of 15a and anti-PD-L1 (n  =  6). * p < 0.05; ** p < 0.01; *** p < 0.001.

# Table 1, antibody information

| **Primary antibody** | **Source** |
| --- | --- |
| Phospho-Stat1(Tyr701) (58D6) Rabbit mAb | CST, 9167 |
| Stat1 (D1K9Y) Rabbit mAb | CST, 14994 |
| p21Waf1/Cip1 (12D1) Rabbit mAb | CST, 2947 |
| PD-L1(E1L3N®) XP® Rabbit mAb | CST, 13684 |
| MDA-5 (D74E4) Rabbit mAb | CST, 5321 |
| RIG-I (D33H10) Rabbit mAb | CST, 4200 |
| HDAC1 (D5C6U) XP Rabbit mAb | CST, 34589 |
| Cleaved-PARP(Asp214) Antibody | CST, 9541 |
| MAVS (E8Z7M) Rabbit mAb | CST, 83000 |
| Actin antibody (Mouse mAb) | Beyotime, AF0003 |
| Cleaved Caspase-3 (Asp175) (5A1E) Rabbit mAb | CST, 9664 |
| Cleaved Caspase-8 (Asp391) (18C8) Rabbit mAb | CST, 8592 |
| Cleaved Caspase-7 (Asp198) Antibody | CST, 9491 |
| Cleaved Caspase-9 (Asp315) (D8I9E) Rabbit mAb | CST, 20750 |
| Histone H3 (D1H2) XP Rabbit mAb | CST, 4499 |
| p27 Kip1 (D69C12) XP® Rabbit mAb | CST, 3686 |
| Acetyl-Histone H3 (Lys27) (D5E4) XP Rabbit mAb | CST, 8173 |
| DNMT1 (D63A6) XP® Rabbit mAb | CST, 5032 |
| Human CCL5/RANTES Antibody | R&D, [AF-278-NA](https://www.rndsystems.com/cn/products/human-ccl5-rantes-antibody_af-278-na) |
| RecombinantAnti-CXCL10 antibody [EPR20764] | Abcam, ab214668 |

# Table 2, primers sequences

| **Primer** | **5’ to 3’** |
| --- | --- |
| ERVL, F | ATATCCTGCCTGGATGGGGT |
| ERVL, R | GAGCTTCTTAGTCCTCCTGTGT |
| MLT1B, F | TGCCTGTCTCCAAACACAGT |
| MLT1B, R | TACGGGCTGAGCTTGAGTTG |
| MLT1C627, F | TGTGTCCTCCCCCTTCTCTT |
| MLT1C627, R | GCCTGTGGATGTGCCCTTAT |
| MER21C, F | GGAGCTTCCTGATTGGCAGA |
| MER21C, R | ATGTAGGGTGGCAAGCACTG |
| MER4D, F | CCCTAAAGAGGCAGGACACC |
| MER4D, R | TCAAGCAATCGTCAACCAGA |
| ISG15, F | GCCTCAGCTCTGACACC |
| ISG15, R | CGAACTCATCTTTGCCAGTACA |
| OASL, F | GCAGAAATTTCCAGGACCAC |
| OASL, R | CCCATCACGGTCACCATTG |
| RIG-I, F | CCAGCATTACTAGTCAGAAGGAA |
| RIG-I, R | CACAGTGCAATCTTGTCATCC |
| IFNL1, F | GAAGACAGGAGAGCTGCAAC |
| IFNL1, R | GGTTCAAATCTCTGTCACCACA |
| IFNL2, F | TCCAGTCACGGTCAGCA |
| IFNL2, R | CAGCCTCAGAGTGTTTCTTCT |
| MAVS, F | AGGAGACAGATGGAGACACA |
| MAVS, R | CAGAACTGGGCAGTACCC |
| MDA5, F | CACTTCCTTCTGCCAAACTTG |
| MDA5, R | GAGCAACTTCTTTCAACCACAG |
| STAT1, F | CGGCTGAATTTCGGCACCT |
| STAT1, R | CAGTAACGATGAGAGGACCCT |
| IFNα (IFNA), F | AATGACAGAATTCATGAAAGCGT |
| IFNα (IFNA), R | GGAGGTTGTCAGAGCAGA |
| IFNβ (IFNB), F | GCCATCAGTCACTTAAACAGC |
| IFNβ (IFNB), R | GAAACTGAAGATCTCCTAGCCT |
| HLA-A, F | TCAGATAGAAAAGGAGGGAGTTACA |
| HLA-A, R | ACAAGCTGTGAGGGACACAT |
| HLA-B, F | CCTGAGATGGGAGCCGTCTT |
| HLA-B, R | CTCCGATGACCACAACTGCT |
| CCL5, F | CCAGCAGTCGTCTTTGTCAC |
| CCL5, R | CTCTGGGTTGGCACACACTT |
| CXCL9, F | AAGACCTTAAACAATTTGCCCC |
| CXCL9, R | TGCTGAATCTGGGTTTAGACAT |
| CXCL10, F | GTGGCATTCAAGGAGTACCTC |
| CXCL10, R | TGATGGCCTTCGATTCTGGATT |
| OAS2, F | GCTTCCGACAATCAACAGCCAAG |
| OAS2, R | CTTGACGATTTTGTGCCGCTCG |
| GAPDH, F | GGAGCGAGATCCCTCCAAAAT |
| GAPDH, R | GGCTGTTGTCATACTTCTCATGG |
| IFI44, F | ATGGCAGTGACAACTCGTTTG |
| IFI44, R | TCCTGGTAACTCTCTTCTGCATA |
| IFI44L, F | GAGCACAGAAATAGGCTTCTAGC |
| IFI44L, R | TGGTATCAGACCCCACTACGG |
| mRig-i, F | GAGCCAGCGGAGATAACAATA |
| mRig-i, R | CCCACGTACTCATAGAGAATGAC |
| mMavs, F | TTACCCAGCAACTCAGTGTATG |
| mMavs, R | CTGTTCCTCTCAGGTGGTATTG |
| mβ-actin, F | GCTTCTTTGCAGCTCCTTCGT |
| mβ-actin, R | CGTCATCCATGGCGAACTG |
| mIfnβ1, F | GGAAAGATTGACGTGGGAGAT |
| mIfnβ1, R | CAGGCGTAGCTGTTGTACTT |
| mMda5, F | ATGGACGCAGATGTTCGTGG |
| mMda5, R | TCCCTTCTCGAAGCAAGTGTC |
| mIfnα15, F | GCTTTCCTGATGACCCTGCT |
| mIfnα15, R | GCTGGGTCAGCTCTTGTAGG |
| mIl23a, F | AAGGAGGTGGATAGGGGGTC |
| mIl23a, R | AGGGCAACAGCCATAGCATT |
| mIfnl3, F | TACACAGCTTCAGGCCACAG |
| mIfnl3, R | CCGGAGGAGCAGTTGAAACA |
| mIfnl2, F | AAGGTCTGGGAGAACATGACTG |
| mIfnl2, R | CTCCATTGGCCACACACTTG |
| mStat1, F | CGCGCATGCAACTGGCATATAACT |
| mStat1, R | AAGCTCGAACCACTGTGACATCCT |

# Table 3, instantaneous sequences

| **Gene** | **Genetic sequence** |
| --- | --- |
| hHDAC1, #1 | GCGACTGTTTGAGAACCTT |
| hHDAC1, #2 | AGGCGGTGGTTACACCATT |
| hDNMT1, #1 | GGAAGAAGAGUUACUAUAA |
| hDNMT1, #2 | UUUGAUGUCAGUCUCAUUGGG |
| hMDA5, #1 | GUUAUAGUUCUUGUCAAUATT |
| hMDA5, #2 | GTGCATGAGGGAGGAACTG |
| hRIG-I, #1 | GAGGUGCAGUAUAUUCAGG |
| hRIG-I, #2 | AAUUCAUCAGAGAUAGUCATT |
| hMAVS, #1 | CACAGGGUCAGUUGUAUCUTT |
| hMAVS, #2 | CCACCUUGAUGCCUGUGAA |
| hMAVS, #3 | GGGUUCUUCUGAGAUUGAATT |
| hIFNAR2, #1 | GGUUAAGAACUGUGCAAAUTT |
| hIFNAR2, #2 | GCCUGAUUACACAGAUGAATT |
| mMavs, #1 | CCAGAUUGGUCCCAGUAAATT |
| mMavs, #2 | GCCACCUGUUUCAGUACUATT |

# Table 4, stable rotation sequences

| shMDA5, #1, F | CCGGCGCAAGGAGTTCCAACCATTTCTCGAGAAATGGTTGGAACTCCTTGCGTTTTTG |
| --- | --- |
| shMDA5, #1, R | AATTCAAAAACGCAAGGAGTTCCAACCATTTCTCGAGAAATGGTTGGAACTCCTTGCG |
| shMDA5, #2, F | CCGGCCAACAAAGAAGCAGTGTATACTCGAGTATACACTGCTTCTTTGTTGGTTTTTG |
| shMDA5, #2, R | AATTCAAAAACCAACAAAGAAGCAGTGTATACTCGAGTATACACTGCTTCTTTGTTGG |
| shRIG-I, #1, F | CCGGCCATGTGAAGTACAAGACATTCTCGAGAATGTCTTGTACTTCACATGGTTTTTG |
| shRIG-I, #1, R | AATTCAAAAACCATGTGAAGTACAAGACATTCTCGAGAATGTCTTGTACTTCACATGG |
| shRIG-I, #2, F | CCGGGCAAGATCTTACTCAGAGATTCTCGAGAATCTCTGAGTAAGATCTTGCTTTTTG |
| shRIG-I, #2, R | AATTCAAAAAGCAAGATCTTACTCAGAGATTCTCGAGAATCTCTGAGTAAGATCTTGC |
| shMAVS, #1, F | CCGGCCAGAGGAGAATGAGTATAAGCTCGAGCTTATACTCATTCTCCTCTGGTTTTTG |
| shMAVS, #1, R | AATTCAAAAACCAGAGGAGAATGAGTATAAGCTCGAGCTTATACTCATTCTCCTCTGG |
| shMAVS, #2, F | CCGGTTTACCAAGGGTTGGATATATCTCGAGATATATCCAACCCTTGGTAAATTTTTG |
| shMAVS, #2, R | AATTCAAAAATTTACCAAGGGTTGGATATATCTCGAGATATATCCAACCCTTGGTAAA |
| shIFNLR1, #1, F | CCGGCCTACATTGAACCACCTTCTTCTCGAGAAGAAGGTGGTTCAATGTAGGTTTTTG |
| shIFNLR1, #1, R | AATTCAAAAACCTACATTGAACCACCTTCTTCTCGAGAAGAAGGTGGTTCAATGTAGG |
| shIFNLR1, #2, F | CCGGGCCGGAAACAAGACCCTATTTCTCGAGAAATAGGGTCTTGTTTCCGGCTTTTTG |
| shIFNLR1, #2, R | AATTCAAAAAGCCGGAAACAAGACCCTATTTCTCGAGAAATAGGGTCTTGTTTCCGGC |
| shIFNAR2, #1, F | CCGGGCAGTAATAAAGTCTCCCTTACTCGAGTAAGGGAGACTTTATTACTGCTTTTTG |
| shIFNAR2, #1, R | AATTCAAAAAGCAGTAATAAAGTCTCCCTTACTCGAGTAAGGGAGACTTTATTACTGC |
| shIFNAR2, #2, F | CCGGCGCCTGATTACACAGATGAATCTCGAGATTCATCTGTGTAATCAGGCGTTTTTG |
| shIFNAR2, #2, R | AATTCAAAAACGCCTGATTACACAGATGAATCTCGAGATTCATCTGTGTAATCAGGCG |
