# Supplementary figures and images for: Dual inhibitors of DNMT and HDAC induce viral mimicry to induce antitumour immunity in breast cancer
Source: Cell Death Discov. 2024 Mar 15;10:143. doi: 10.1038/s41420-024-01895-7 (PMC10943227; doi:10.1038/s41420-024-01895-7)

Figure 2

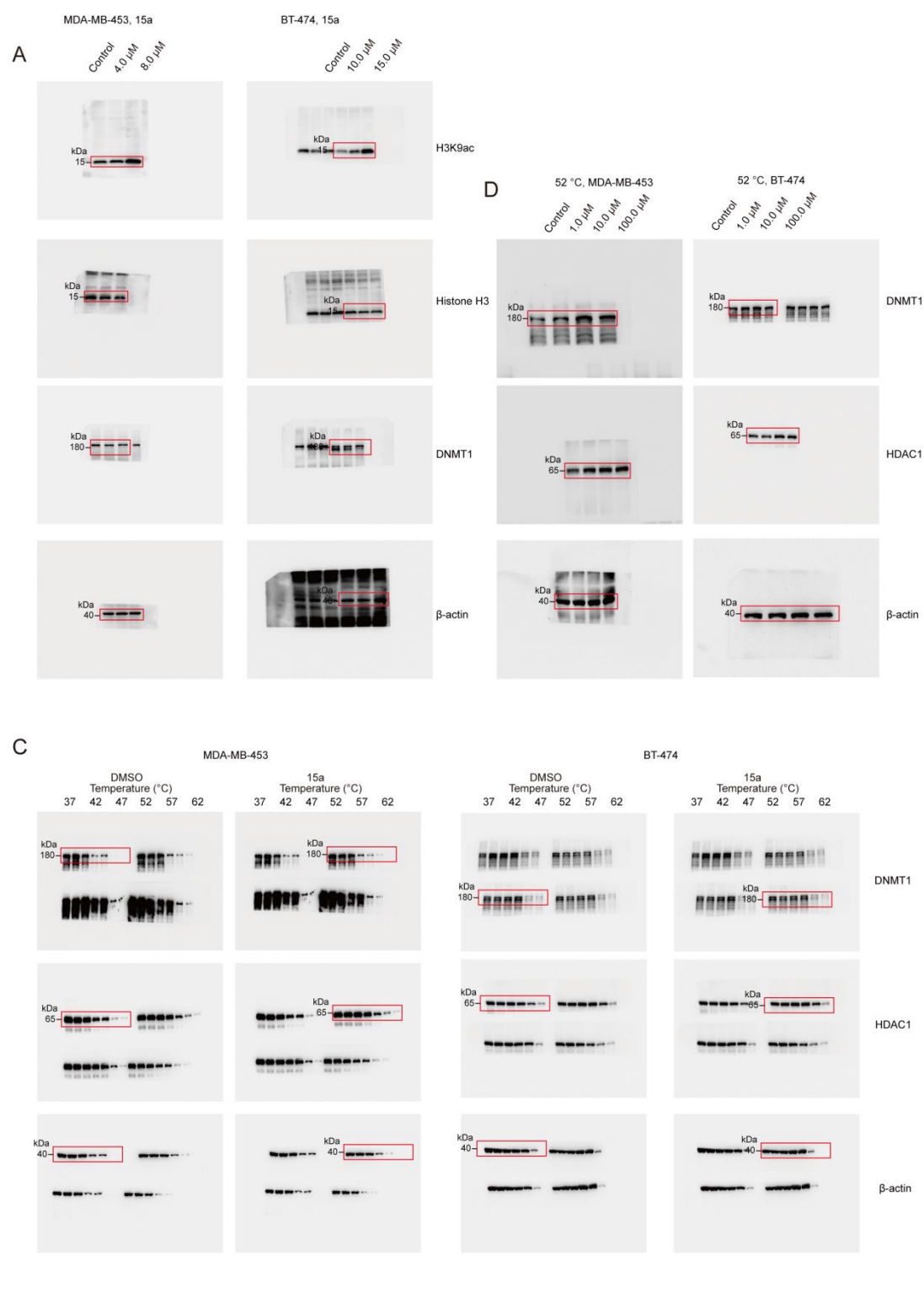

Figure 3

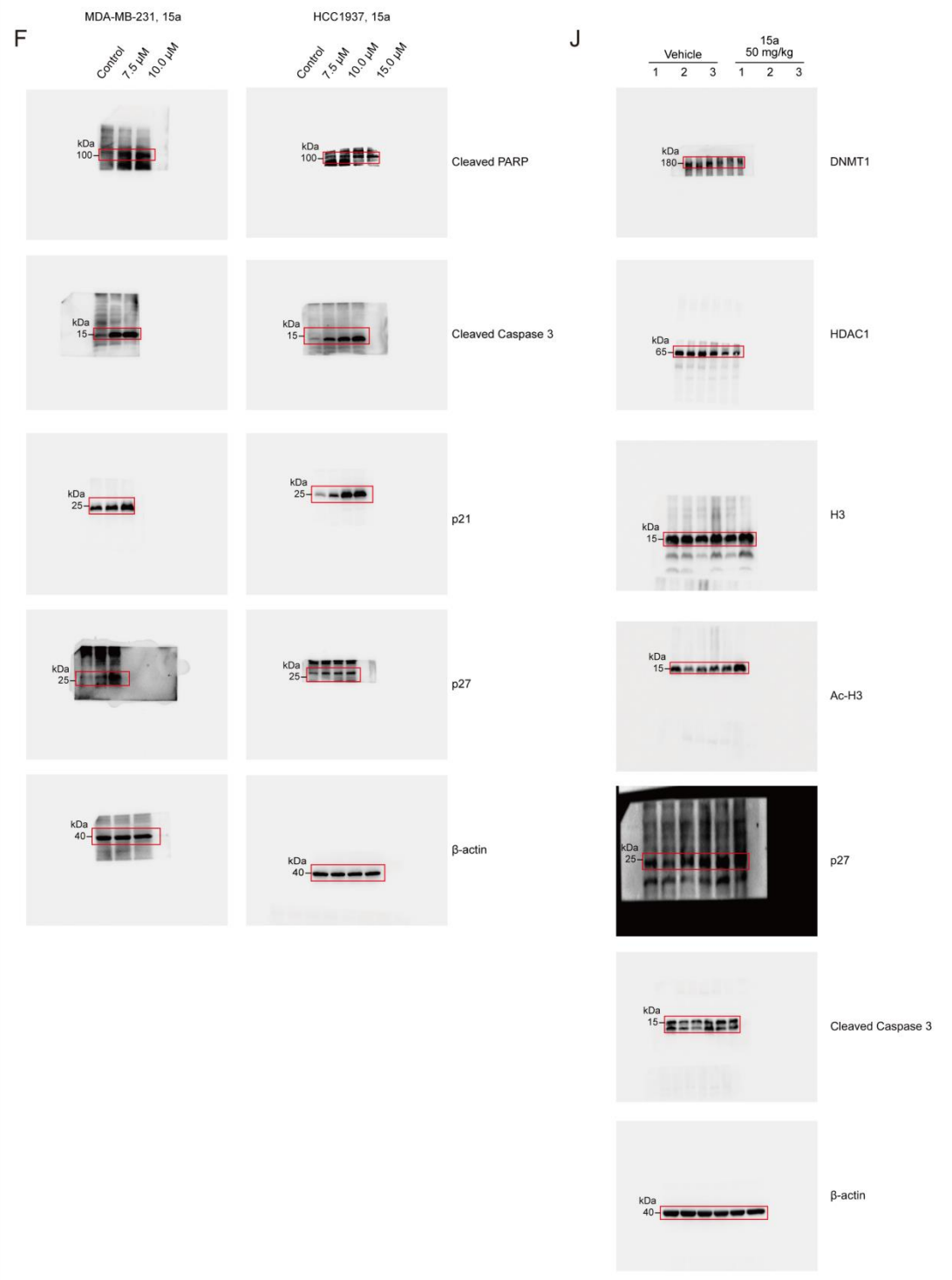

Figure 4

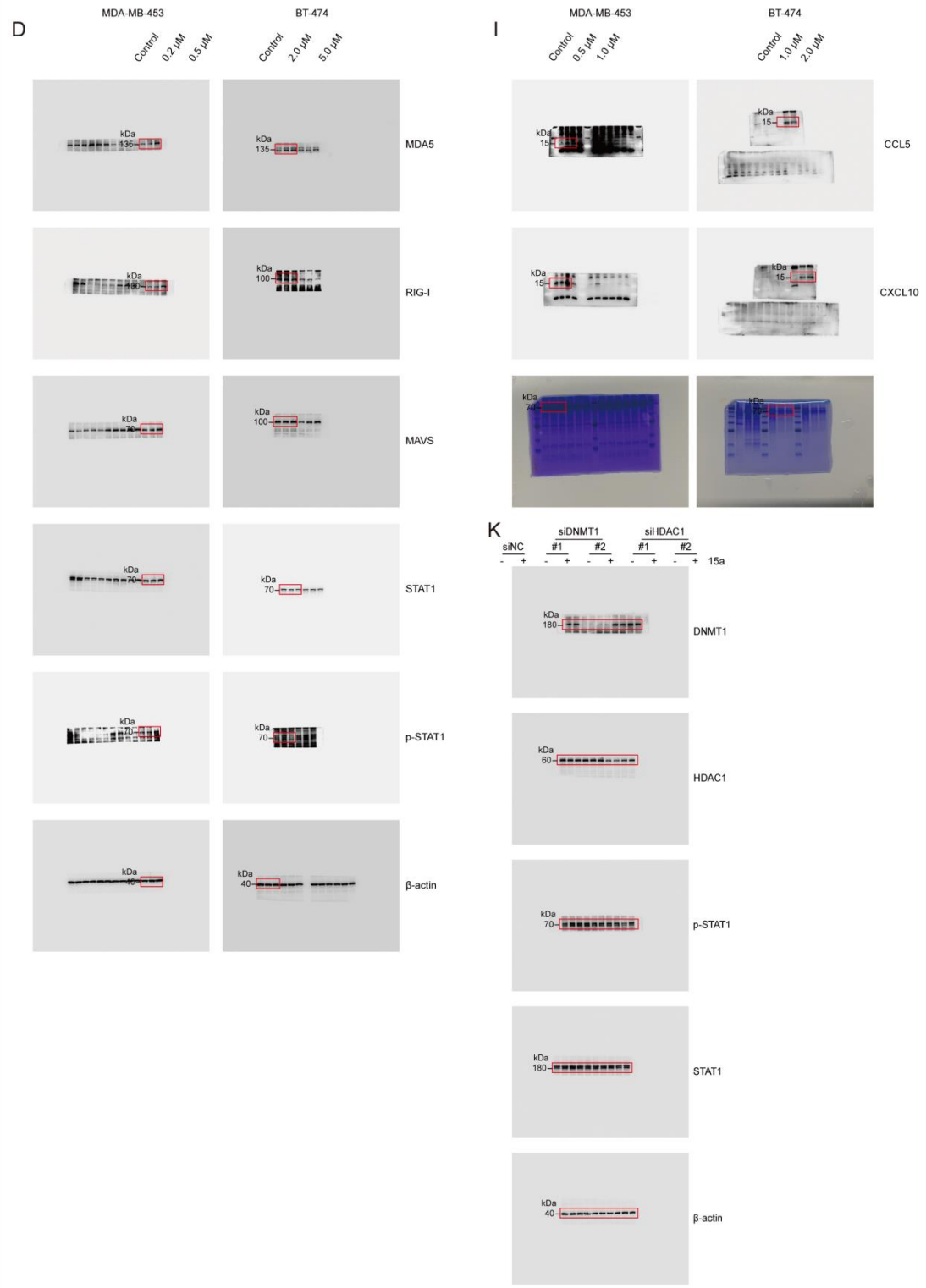

Figure 5

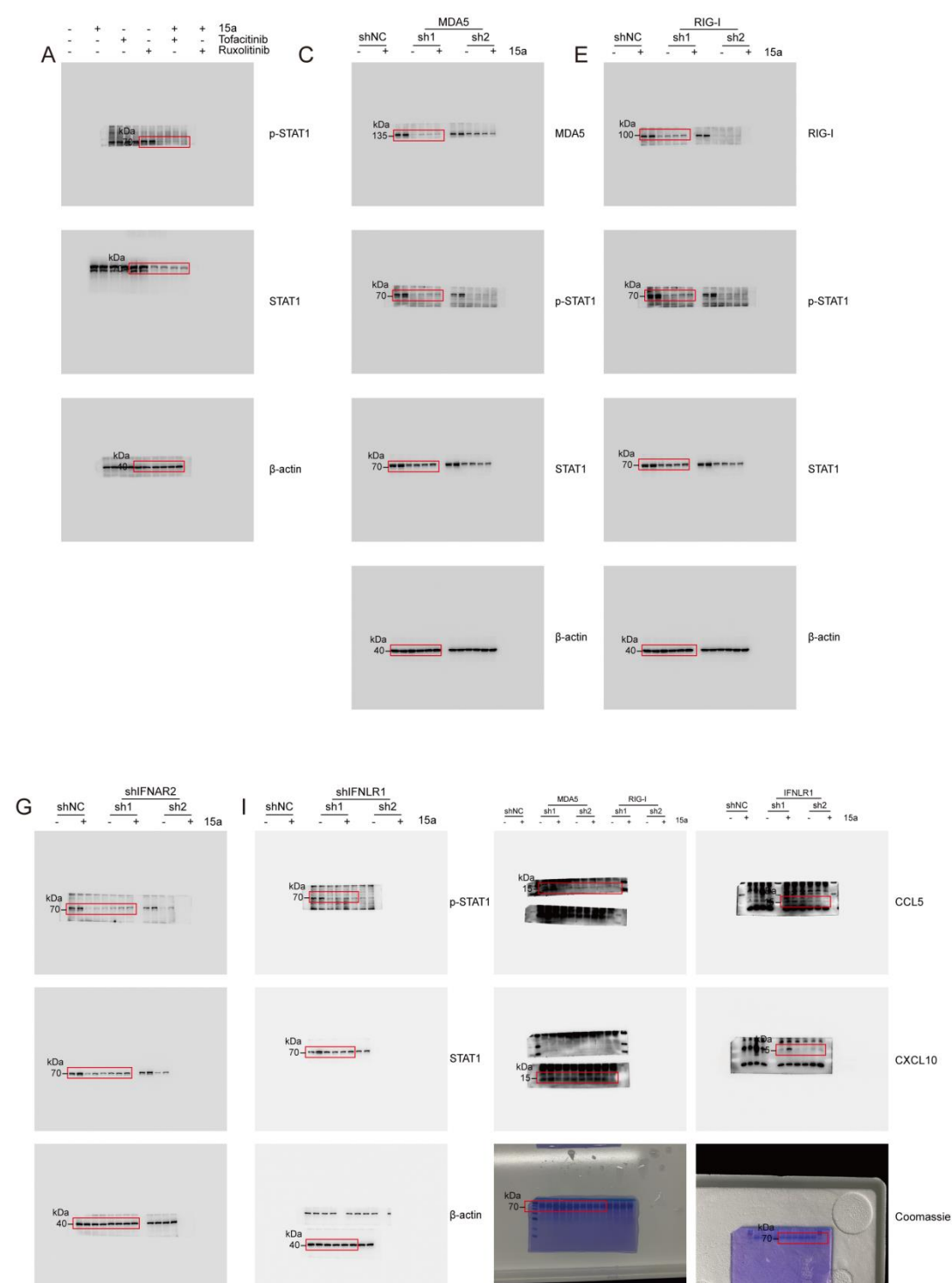

Figure 6

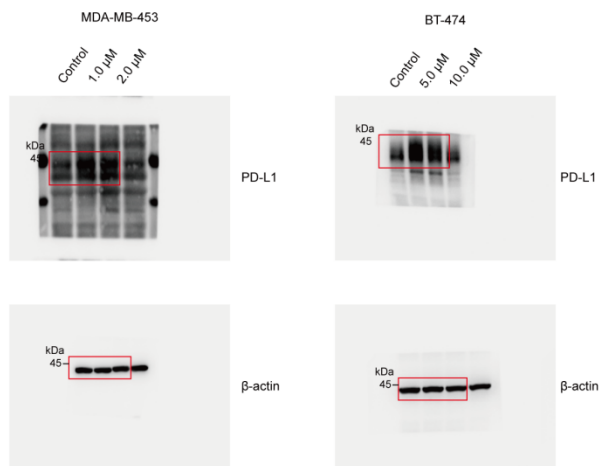

Figure S1

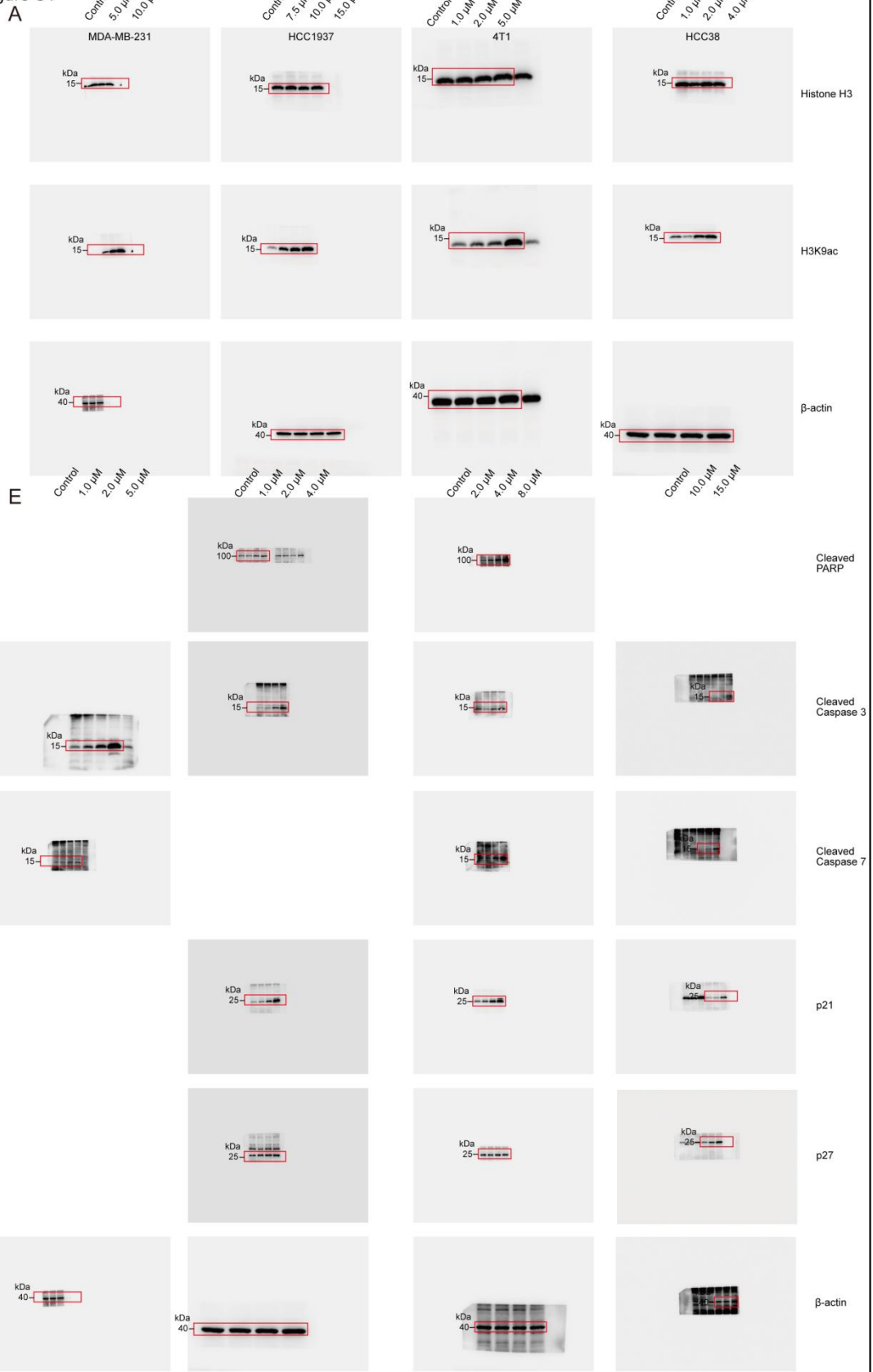

A

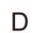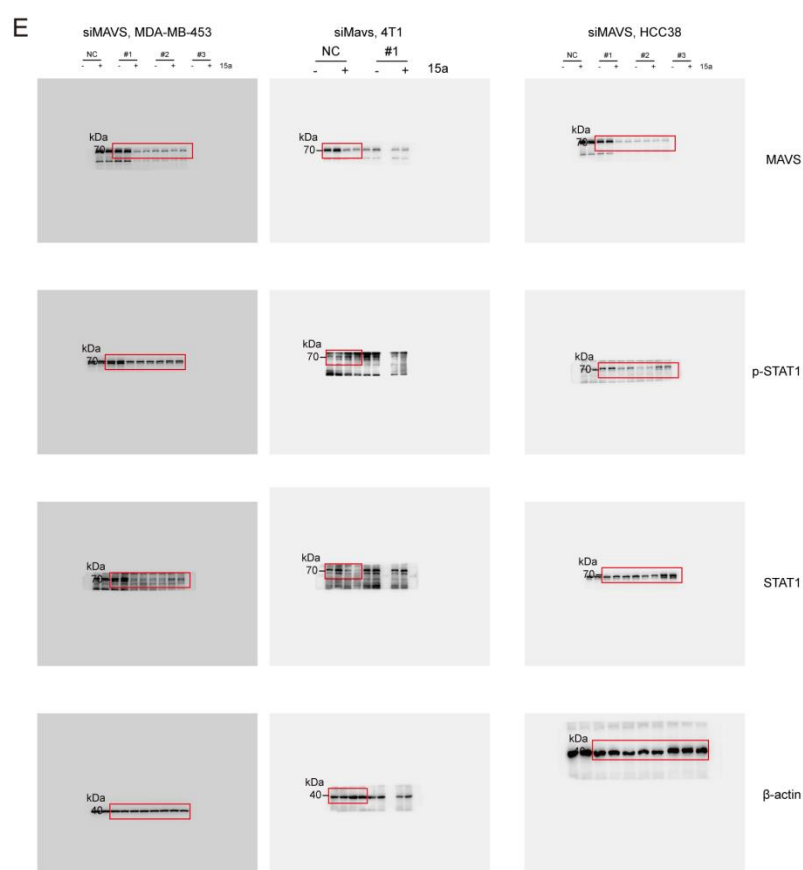

Figure S2-1

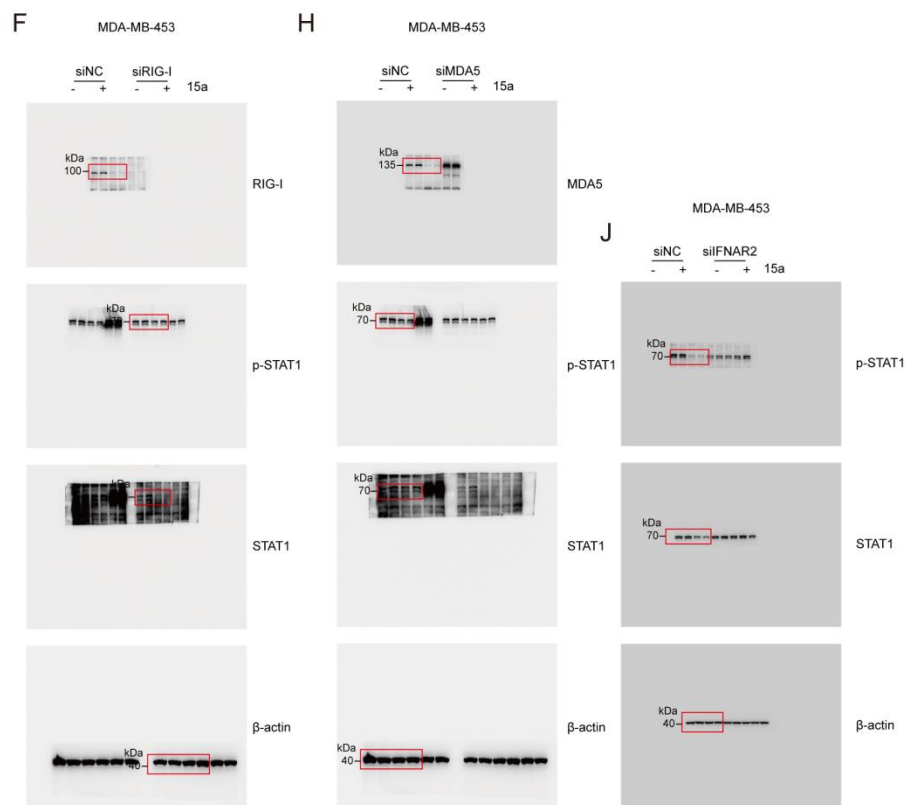

Supplement: Supplementary file 2 — Original Data File [file 41420_2024_1895_MOESM2_ESM.pdf]
